# Catalogue of the type material of Scarabaeoidea (Coleoptera) deposited in the Research Institute of Evolutionary Biology, Tokyo, Japan

**DOI:** 10.3897/zookeys.958.52799

**Published:** 2020-08-11

**Authors:** Naoki Kaneko, Kaoru Wada

**Affiliations:** 1 Laboratory of Entomology, Tokyo University of Agriculture, 1737 Funako, Atsugi, Kanagawa, 243-0034, Japan Tokyo University of Agriculture Tokyo Japan; 2 School of Science and Engineering, Meisei University, 2-1-1 Hodokubo, Hino, Tokyo 191-8506, Japan Meisei University Tokyo Japan

**Keywords:** Allotype, Geotrupidae, holotype, Lucanidae, neotype, paratype, Scarabaeidae, Trogidae

## Abstract

A detailed catalogue of the type material of the Scarabaeoidea housed in the Research Institute of Evolutionary Biology, Tokyo, Japan is listed. The catalogue includes the data of the type material of four families and 111 species.

## Introduction

In this report we present a catalogue of the type material of Scarabaeoidea housed in the Research Institute of Evolutionary Biology, Tokyo, Japan (RIEB). The beginning of the RIEB collection dates back to 1950, when the first natural objects were deposited at the Research Institute of Thremmatology of the Tokyo University of Agriculture (RIT) established that year. RIT was transformed into an incorporated foundation in 1974 and renamed Research Institute of Evolutionary Biology (Yamamoto 2000). The Lepidoptera collection in the RIEB was listed in detail by Aoki et al. (2005, 2008) and Yamaguchi et al. (2006, 2008, 2009, 2011, 2017), but the Coleoptera collection has never been catalogued. This creates a problem because well-organized museum collections and published type specimen catalogues are very important to facilitate taxonomic and systematic investigations of animals.

The majority of the types of Scarabaeidae deposited in the RIEB originate from the collection of Mr Yoshikazu Miyake (1926–2003), a well-known Japanese amateur specialist on Scarabaeidae, who was also the author of those taxa. A part of types of Miyake’s species is also housed in the National Museum of Nature and Science, Tokyo (NMNS) and the Nagaoka Municipal Science Museum, Niigata (NMSM). Unfortunately, some of his types are missing.

In addition, other researchers that contributed to the Scarabaeoidea type collection in RIEB are: Dirk Ahrens, Aleš Bezděk, Toshiyuki Ichikawa, Takeshi Itoh (Takeshi Matsumoto), Kimio Masumoto, Sizumu Nomura, Teruo Ochi, and Kaoru Wada.

## Materials and methods

The scientific names of higher taxa in this catalogue follow Scholtz and Grebennikov (2016). The structure of each entry is as follows:

– original combination of the taxon name;

– original combination and spelling of the taxon name, followed by the author, year of description, and pagination;

– type material, number of specimens (including sex, if known), and exact label data. A double slash ‘//’ indicates separate labels and single slash ‘/’ indicates lines within each label. The words in Japanese or Chinese were transcribed into Roman alphabet. Paratypes with discrepancies between collection data on the label and data quoted in the original description are indicated by ‘ [[]]’;

– remarks on types condition (given only for holotypes and neotypes);

– current taxonomic status;

– remarks, if any.

The majority of holotypes and neotypes were photographed with a Nikon D7200 camera using a Nikon AF-S DX Micro NIKKOR 40 mm f/2.8 G lens, and some types were photographed with a KEYENCE VHX-1000 Digital Microscope.

## Catalogue

### Family Geotrupidae

#### Subfamily Geotrupinae

**Genus *Sinogeotrupes***

##### Sinogeotrupes taiwanus

Taxon classificationAnimaliaColeopteraGeotrupidae

Miyake & Yamaya

966AF9F9-8E1F-5A78-BF35-005255BDABBB

Sinogeotrupes taiwanus Miyake & Yamaya, 1995: 32−34.

###### Note.

The following paratypes are deposited in RIEB (ex coll. Y. Miyake):

###### Paratypes.

3 exs.: 1 ex. ‘Wusha / –VI–1941 / R. Boloudo // Paratype / Sinogeotrupes taiwanus / Y. MIYAKE et YAMAYA, 1995’. 1 ex. ‘Wu sha / Formosa / 21. Viii. 1941 / Col. Bumua Tin // Geotrupes / subshiatelluo FAIRMAIR / DET. Y. MIYAKE // Paratype / Sinogeotrupes taiwanus / Y. MIYAKE et YAMAYA, 1995’. 1 ex. ‘台湾= taiwan 台中= taichû / 関刀山= guandaoshan / 1993. V. 31 // Paratype / Sinogeotrupes taiwanus / Y. MIYAKE et YAMAYA, 1995’.

###### Current status.

Phelotrupes (Sinogeotrupes) taiwanus (Miyake and Yamaya, 1995), see Nikolajev et al. (2016).

###### Remark.

In addition to the paratypes mentioned above, the following specimens labeled as paratypes are not designated in the original description: 3 exs. ‘Tattaka / Formosa / 10. Vi. 1965 / T. Shirozu // Paratype / Sinogeotrupes taiwanus / Y. MIYAKE et YAMAYA, 1995’. 1 ex. ‘(FORMOSA) / Mt Rata / Vi–Vii–1972 / L. F, Hang // Paratype / Sinogeotrupes taiwanus / Y. MIYAKE et YAMAYA, 1995’. 1 ex. ‘(FORMOSA) / Mt Rata / –Vii–1972 / L. F, Hang // Paratype / Sinogeotrupes taiwanus / Y. MIYAKE et YAMAYA, 1995’. 2 exs. ‘ [Taiwan] / 台中= taichû 関刀山= guandaoshan / 3–VI–1993 // Paratype / Sinogeotrupes taiwanus / Y. MIYAKE et YAMAYA, 1995’. 1 ♂ ‘ [Taiwan] / 台中= taichû 関刀山= guandaoshan / 3–VI–1993 // Paratype / Sinogeotrupes taiwanus / Y. MIYAKE et YAMAYA, 1995’. 3 exs. ‘Paratype / Sinogeotrupes taiwanus / Y. MIYAKE et YAMAYA, 1995’.

### Family Lucanidae

#### Subfamily Lucaninae

**Genus *Aegus***

##### Aegus laevicollis nakanei

Taxon classificationAnimaliaColeopteraLucanidae

Ichikawa & Imanishi

21CE787E-F0F2-55A9-AC6C-39D33669E543

Aegus laevicollis nakanei Ichikawa & Imanishi, 1976: 9.

###### Note.

The following paratype is deposited in RIEB (ex coll. Y. Kusui):

###### Paratype.

1 ex.: [[1 ♀ ‘Mt. Yonahadake, / Kunigami-son, / Is. Okinawa-Hontō, / 28. VI. 1972 (the date given in the original description is 16–17. VI. 1970), / HEIKICHI IRIE leg., // KUSUI // Paratype’]].

###### Current status.

*Aegus nakanei nakanei* Ichikawa and Imanishi, 1976, see Huang and Chen (2017).

### Family Scarabaeidae

#### Subfamily Aphodiinae

**Genus *Aphodius***

##### Aphodius (Agoliinus) tanakai

Taxon classificationAnimaliaColeopteraAphodiidae

Masumoto

CF4CEE41-C8E6-5494-8E87-B13695D5B206

Aphodius (Agoliinus) tanakai Masumoto, 1981: 73−74.

###### Note.

The following paratype is deposited in RIEB (ex coll. K. Masumoto):

###### Paratype.

1 ex.: ‘Nikko / Date: 23 V 1981 / K. MASUMOTO leg. // Paratype / Aphodius (Agr) / tanakai MASUMOTO’.

###### Current status.

*Agoliinus tanakai* (Masumoto, 1981), see Dellacasa et al. (2016).

##### Aphodius (Phaeaphodius) himalorectus

Taxon classificationAnimaliaColeopteraAphodiidae

Ahrens & Stebnicka

3D25AD27-F087-541C-BBF8-7D063B0A2768

Aphodius (Phaeaphodius) himalorectus Ahrens & Stebnicka, 1997: 12−13.

###### Note.

The following paratypes are deposited in RIEB (ex coll. D. Ahrens):

###### Paratypes.

2 exs.: ‘Phalante 20. 3. / unt. Nebelwaldstufe / 2100–2300 m // PARATYPUS / Aphodius (Phaeaphodius) himalorectus sp. n. / det. D. AHRENS 1994’.

###### Current status.

*Phaeaphodius himalorectus* (Ahrens and Stebnicka, 1997), see Dellacasa et al. (2016).

##### Aphodius (Sinodiapterna) zeni

Taxon classificationAnimaliaColeopteraAphodiidae

Ochi

D196A45C-3FF4-5BDC-93F4-9C9ACFD97AE1

Aphodius (Sinodiapterna) zeni Ochi, 1991: 53−55.

###### Note.

The following paratype is deposited in RIEB (ex coll. T. Ochi):

###### Paratype.

1 ex.: ‘SUNGKAN / C. TAIWAN / 29–V. 1987 / K. LAH // PARATYPE / Aphodius (Sinodiapterna) / zeni / OCHI 1991’.

###### Current status.

*Sinodiapterna zeni* (Ochi, 1991), see Dellacasa et al. (2016).

#### Genus *Rhyparus*

##### Rhyparus kitanoi

Taxon classificationAnimaliaColeopteraAphodiidae

Miyake

77DC2BCD-51B6-5F0F-B2B5-CFCED9337CD1

Figure 1A

Rhyparus kitanoi Miyake, 1982: 65−67.

###### Note.

The holotype and following specimen are deposited in RIEB (ex coll. Y. Miyake):

###### Holotype

**Figure 1.**
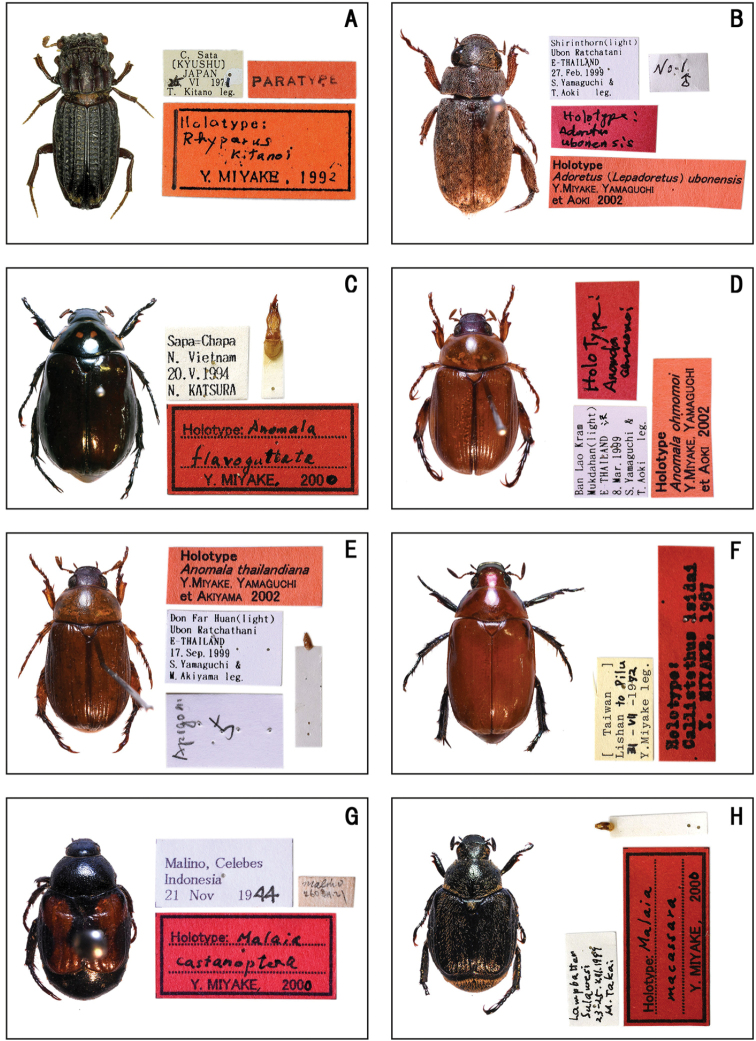
Habitus of holotype specimens. **A***Rhyparus kitanoi* (Miyake) **B**Adoretus (Lepadoretus) ubonensis (Miyake, Yamaguchi et Aoki) **C***Anomala flavoguttata* Miyake **D***Anomala ohmomoi* Miyake, Yamaguchi et Aoki **E***Anomala thailandiana* Miyake, Yamaguchi et Akiyama **F***Callistethus isidai* Miyake **G***Malaia castanoptera* Miyake **H***Malaia macassara* Miyake.

**(♂).** ‘C, Sata /〔KYUSHU〕/ Japan / 25, VI 1971 / T. Kitano leg. // PARATYPE // Holotype: / Rhyparus / kitanoi / Y. MIYAKE, 1992’. (Fig. 1A)

###### Type condition.

The right and left protarsus and the right antenna of the holotype are missing.

###### Current status.

*Rhyparus kitanoi kitanoi* Miyake, 1982, see Ochi (2001).

###### Remark.

In addition to the holotype mentioned above, the following specimen labeled as paratype is not designated in the original description: 1 ex.: ‘Sesonoura / Is. Shimokoshiki / Kagoshima-pref. / 18. VI. 1982 / S. Imasaka leg. // Paratype: Rhyparus / kitanoi / Y. MIYAKE, 1992’.

#### Subfamily Scarabaeinae

**Genus *Caccobius***

##### Caccobius bawangensis

Taxon classificationAnimaliaThelebolalesThelebolaceae

Ochi, Kon & Kikuta

F7535001-A039-5B7D-B745-B4A47296800D

Caccobius bawangensis Ochi, Kon & Kikuta, 1997: 262−264.

###### Note.

The following paratype is deposited in RIEB (ex coll. T. Ochi):

###### Paratype.

1 ex.: ‘Mt. Bawang / Sarawak S. / VIII. 1991 / M. Kon Coll. // PARATYPE / Caccobius / bawangensis OCHI, KON and KIKUTA’.

###### Current status.

Valid species.

#### Genus *Copris*

##### Copris (Microcopris) mindorensis

Taxon classificationAnimaliaColeopteraScarabaeidae

Ochi

6DF1E607-BEF2-52B9-94CA-57E10473BA39

Copris (Microcopris) mindorensis Ochi, 1992: 13.

###### Note.

The following paratype is deposited in RIEB (ex coll. T. Ochi):

###### Paratype.

1 ex.: ‘MT. HALKON / IS. MINDRO / 28–XII. 1988 / D. MOHAGAN // PARATYPE / Copris / (Microcopris) / mindorensis / OCHI, 1992.

###### Current status.

Valid species.

#### Genus *Onthophagus*

##### Onthophagus setchan

Taxon classificationAnimaliaColeopteraScarabaeidae

Masumoto

F4B7452C-5C3F-551A-93E0-4CA97A3BADF9

Onthophagus setchan Masumoto, 1984: 80−81.

###### Note.

The following paratype is deposited in RIEB (ex coll. K. Masumoto):

###### Paratype.

1 ex.: ‘FENGCHIFU / FORMOSA / 7–VII. 74. / T. OCHI // PARATYPE / Onthophagus / setchan / MASUMOTO (1984)’.

###### Current status.

Valid species.

##### Onthophagus (Parascatonomus) acuticollissakishimanus

Taxon classificationAnimaliaColeopteraScarabaeidae

Nomura

71EEA96C-0871-54B2-8640-A8C6227EBA91

Onthophagus (Parascatonomus) acuticollis sakishimanus Nomura, 1976a: 29.

###### Note.

The following paratypes are deposited in RIEB (ex coll. S. Nomura):

###### Paratypes.

6 exs.: 3 ♂ ‘Kanpira / Iriomote Is. / Yaeyama Iss. / 12. VII. 1975 / R. YANO leg. // ♂ // Onthophagus (P.) / acuticollis / sakishimanus / NOMURA (1976)’. 3 ♀ ‘Kanpira / Iriomote Is. / Yaeyama Iss. / 12. VII. 1975 / R. YANO leg. // ♀ // Onthophagus (P.) / acuticollis / sakishimanus / NOMURA (1976)’.

###### Current status.

Parascatonomus (Necramator) acuticollis sakishimanus (Nomura, 1976), see Ochi and Kon (2017).

##### Onthophagus (Parascatonomus) murasakianus

Taxon classificationAnimaliaColeopteraScarabaeidae

Nomura

B86813CD-6881-58EB-A9F2-4A6A0912486F

Onthophagus (Parascatonomus) murasakianus Nomura, 1976a: 30.

###### Note.

The following paratype is deposited in RIEB (ex coll. S. Nomura):

###### Paratype.

1 ex.: ‘Kanpira, / Iriomote Is. / Yaeyama Iss. / 12. VII 1975 / R. YANO leg. // PARATYPE / Onthophagus (P.) / murasakianus / NOMURA (1976)’.

###### Current status.

Parascatonomus (Necramator) murasakianus (Nomura, 1976), see Ochi and Kon (2017).

##### Onthophagus (Parascatonomus) takedai

Taxon classificationAnimaliaColeopteraScarabaeidae

Ochi & Araya

7562CF62-422A-5F5B-BD6B-0FC6CDFCC2EA

Onthophagus (Parascatonomus) takedai Ochi & Araya, 1992: 99−100.

###### Note.

The following paratype is deposited in RIEB (ex coll. T. Ochi):

###### Paratype.

1 ex.: ‘QUESON. N. P / IS. LUZON / 10–VIII. 1981 / K. SUGINO // PARATYPE / Onthophagus / (Parascatonomus) / takedai / OCHI et ARAYA, 1992’.

###### Current status.

Parascatonomus (Granulidorsum) takedai (Ochi et Araya, 1992), see Ochi and Kon (2017).

##### Onthophagus (Proagoderus) watanabei

Taxon classificationAnimaliaColeopteraScarabaeidae

Ochi & Kon

E282C728-2248-58F9-9300-AB84DB9B23F1

Onthophagus (Proagoderus) watanabei Ochi & Kon, 2002: 306−311.

###### Note.

The following paratype is deposited in RIEB (ex coll. T. Ochi):

###### Paratype.

1 ex.: ‘SEPIROK / N. BORNEO / 4–VII. 1985 / M. KON // PARATYPE / Onthophagus / (Proagoderus) / watanabei / OCHI and KON. 2002’.

###### Current status.

*Proagoderus watanabei* (Ochi et Kon, 2002), see Philips (2016).

##### Onthophagus (Strandius) hiurai

Taxon classificationAnimaliaColeopteraScarabaeidae

Ochi

0955D712-4CC3-5698-B6B9-49F9285CFC80

Onthophagus (Strandius) hiurai Ochi, 1984: 63−65.

###### Note.

The following paratype is deposited in RIEB (ex coll. T. Ochi):

###### Paratype.

1 ex.: ‘PILU / Taiwan / 21, VIII, 1979 / K. Sugino leg. // PARATYPE / Onthophagus (St) / hiurai / OCHI (1984)’.

###### Current status.

Onthophagus (Phanaeomorphus) potanini hiurai Ochi, 1984, see Ziani and Bezděk (2016).

#### Subfamily Rutelinae

**Genus *Adoretus***

##### Adoretus (Lepadoretus) ubonensis

Taxon classificationAnimaliaColeopteraRutelidae

Miyake, Yamaguchi & Aoki

6E95AC70-A412-52F8-876E-2E0E31040CDF

Figure 1B

Adoretus (Lepadoretus) ubonensis Miyake, Yamaguchi, Aoki & Akiyama, 2002: 62−63.

###### Note.

The holotype is deposited in RIEB (ex coll. Y. Miyake):

###### Holotype

**(♂).** ‘Shirinthorn (light) / Ubon Ratchatani / E–THAILAND / 27. Feb. 1999 / S. Yamaguchi and / T. Aoki leg. // No. 1 / ♂ // Holotype: / Adoretus / ubonensis // Holotype / Adoretus (Lepadoretus) ubonensis / Y. MIYAKE, YAMAGUCHI / et AOKI 2002’. (Fig. 1B)

###### Type condition.

The left mesotarsus and the left metaleg of the holotype are missing.

###### Current status.

Junior subjective synonym of *Adoretus compressus* (Weber, 1801), see Kobayashi (2018).

#### Genus *Anomala*

##### Anomala flavoguttata

Taxon classificationAnimaliaColeopteraRutelidae

Miyake

F5A76A0A-A44E-57BA-BB1C-1DEA9E3417AA

Figure 1C

Anomala flavoguttata Miyake, 2000: 108−109.

###### Note.

The holotype is deposited in RIEB (ex coll. Y. Miyake):

###### Holotype

**(♂).** ‘Sapa=Chapa / N. Vietnam / 20. V. 1994 / N. KATSURA // Holotype: Anomala / flavoguttata / Y. MIYAKE, 2000’. (Fig. 1C)

###### Type condition.

The aedeagus of the holotype is pinned separately.

###### Current status.

Valid species.

##### Anomala ohmomoi

Taxon classificationAnimaliaColeopteraRutelidae

Miyake, Yamaguchi & Aoki

41E2EE96-E344-5136-98EE-92FE6B007ABC

Figure 1D

Anomala ohmomoi Miyake, Yamaguchi, Aoki & Akiyama, 2002: 58−60.

###### Note.

The holotype and the following paratypes are deposited in RIEB (ex coll. Y. Miyake):

###### Holotype

**(♂).** ‘Ban Lao Kram / Mukdahan (light) / E–THAILAND沢= sawa / 8. Mar. 1999 / S. Yamaguchi and / T. Aoki leg. // Holo Type: / Anomala / ohmomoi // Holotype / *Anomala ohmomoi* / Y. MIYAKE, YAMAGUCHI / et AOKI 2002’. (Fig. 1D)

###### Paratypes.

4 exs.: ‘Ban Lao Kram / Mukdahan (light) / E–THAILAND沢= sawa / 8. Mar. 1999 / S. Yamaguchi and / T. Aoki leg. // Paratype / Anomala ohmomoi / Y. MIYAKE, YAMAGUCHI / et AOKI 2002’.

###### Current status.

Valid species.

##### Anomala thailandiana

Taxon classificationAnimaliaColeopteraRutelidae

Miyake, Yamaguchi & Akiyama

86A394A0-0AAB-5621-B49F-B06FAEC8625E

Figure 1E

Anomala thailandiana Miyake, Yamaguchi, Aoki & Akiyama, 2002: 60−61.

###### Note.

The holotype is deposited in RIEB (ex coll. Y. Miyake):

###### Holotype

**(♂).** ‘Don Far Huan (light) / Ubon Ratchathani / E–THAILAND / 17. Sep. 1999 / S. Yamaguchi and / M. Akiyama leg. [white label, front] // Apigoni / 5 [white label, back] // Holotype / Anomala thailandiana / Y. MIYAKE, YAMAGUCHI / et AKIYAMA 2002’. (Fig. 1E)

###### Type condition.

The aedeagus of the holotype is pinned separately.

###### Current status.

Valid species.

#### Genus *Callistethus*

##### Callistethus isidai

Taxon classificationAnimaliaColeopteraRutelidae

Miyake

88B1C0B4-7A2D-5135-BD6E-3B847A7ADC06

Figure 1F

Callistethus isidai Miyake, 1987: 5−6.

###### Note.

The holotype and the following paratype are deposited in RIEB (ex coll. Y. Miyake):

###### Holotype

**(♂).** ‘ [Taiwan] / Lishan to Pilu / 31–VII–1972 / Y. Miyake leg. // Holotype: / Callistethus isidai / Y. MYAKE, 1987’. (Fig. 1F)

###### Paratype.

1 ex.: ‘ [Taiwan] / Lishan to Pilu / 31–VII–1972 / Y. Miyake leg. // Paratype / Callistethus isidia / Y. MIYAKE, 1987’.

###### Type condition.

The right and left metatarsi of the holotype are missing.

###### Current status.

*Callistethus plagiicollis isidai* Miyake, 1987, see Zorn and Bezděk (2016).

###### Remark.

The habitus photograph in the original description does not agree with the holotype. This is also the case with some other species described by Miyake. Apparently, he did not intend to provide the photographs of the holotypes. Also, as noted below, we were unable to trace some specimens illustrated in the original descriptions.

#### Genus *Kibakoganea*

##### Kibakoganea ohtanii

Taxon classificationAnimaliaColeopteraRutelidae

Miyake & Muramoto

6EF2AFCD-F5E1-5217-B09E-B7C8AEC0FB2E

Kibakoganea ohtanii Miyake & Muramoto, 1992: 22−24.

###### Note.

The following paratypes are deposited in RIEB (ex coll. Y. Miyake):

###### Paratypes.

2 exs.: [[1 ♂, 1 ♀ ‘Tam Dao / N. VIETNAM / 28–30, IV, 1991 (the date given in the original description is 28–30. III. 1991) / M. Fujioka and / R. Muramoto – leg. // PARATYPE / Kibakoganea / ohtanii / Y. MIYAKE et / MURAMOTO, 1992.’]].

###### Current status.

Junior subjective synonym of *Kibakoganea sexmaculata* (Kraatz, 1900), see Miyake (2003).

#### Genus *Malaia*

##### Malaia castanoptera

Taxon classificationAnimaliaColeopteraRutelidae

Miyake

8DD2B02D-0D3F-50C6-AE1B-E6F73F1FEF26

Figure 1G

Malaia castanoptera Miyake, 2000: 112.

###### Note.

The holotype is deposited in RIEB (ex coll. Y. Miyake):

###### Holotype

**(♂).** ‘Malino, Celebes / Indonesia / 21 Nov 1944 // malino / 2603.11.21 // Holotype: Malaia / castanoptera / Y. MIYAKE, 2000’. (Fig. 1G)

###### Paratypes.

7 exs.: 1 ♂, 3 ♀ ‘Malino, Celebes / Indonesia / 21 Nov 1944 // malino / 2603.11.21 // Paratype: Malaia / castanoptera / Y. MIYAKE, 2000’. 2 exs. ‘Malino, Celebes / Indonesia / 21 Nov 1944 // malino / 2603.11.21 // Paratype: Malaia / castanoptera / Y. MIYAKE, 2000’. 1 ex. ‘malino / 2603.11.21 // Paratype: Malaia / castanoptera / Y. MIYAKE, 2000’.

###### Current status.

Valid species.

###### Remark.

The habitus photo in the original description belongs to the female paratype with the label ‘Malino, Celebes / Indonesia / 21 Nov 1944 // malino / 2603.11.21’.

##### Malaia cyanea

Taxon classificationAnimaliaColeopteraRutelidae

Miyake

367CEDFC-08CF-55EA-8F71-404677E01B2A

Malaia cyanea Miyake, 1996: 38−39.

###### Note.

The following paratypes are deposited is RIEB (ex coll. Y. Miyake):

###### Paratypes.

2 exs.: 1 ♂ ‘32 km from / Palopo / Surawesi / 10. VI. 1982 // Paratype: / Malaia cyanea / Y. MIYAKE, 1996’. 1 ♀ ‘Punchak–Lombanan / Mamasa, / S. SULAWESI / 1991. AUG. 10. / N. Kashiwai–leg. // Paratype: / Malaia cyanea / Y. MIYAKE, 1996’.

###### Current status.

Valid species.

##### Malaia macassara

Taxon classificationAnimaliaColeopteraRutelidae

Miyake

267BC972-6F84-5456-B861-E914FFBDC5CA

Figure 1H

Malaia macassara Miyake, 2000: 109−110.

###### Note.

The holotype and the following paratype are deposited in RIEB (ex coll. Y. Miyake):

###### Holotype

**(♂).** ‘Lampbatter / Sulawesi / 23–25. VIII. 1999 / M. Takai // Holotype: Malaia / macassara / Y. MIYAKE, 2000’. (Fig. 1H)

###### Paratype.

1ex.: 1 ♂ ‘Lampbatter / Sulawesi / 23–25. XII. 1999 / M. Takai leg. // Paratype: Malaia / macassara / Y. MIYAKE, 2000’.

###### Type condition.

The left protarsus and metatarsus of the holotype are missing.

###### Current status.

Valid species.

##### Malaia rufofemorata

Taxon classificationAnimaliaColeopteraRutelidae

Miyake

B699423B-6F15-5832-B723-4FB660923D7E

Malaia rufofemorata Miyake, 1996: 40−41.

###### Note.

The following paratypes are deposited in RIEB (ex coll. Y. Miyake):

###### Paratypes.

4 exs.: 1 ♂, 1 ♀ ‘Puncak, Palopo / VI–VII, 1989 / Central SULAWESI // Paratype: Malaia / rufofemorata / Y. MIYAKE, 1996’. 2 ♂ ‘Palu Palolo / VIII. 1990 / Central SULAWESI // Paratype: Malaia / rufofemorata / Y. MIYAKE, 1996’.

###### Current status.

Valid species.

###### Remark.

In addition to the paratypes mentioned above, the following specimens labeled as paratypes are not designated in the original description: 2 ♂, 1 ♀ ‘Palolo, Palu / 01–10/XI. 1990 / native coll. / CELEBES // Paratype: Malaia / rufofemorata / Y. MIYAKE, 1996’.

##### Malaia sulawesi

Taxon classificationAnimaliaColeopteraRutelidae

Miyake

37ADCBA8-9015-51C9-857F-6E8E77A08B24

Malaia sulawesi Miyake, 1996: 41−42.

###### Note.

The following paratypes are deposited in RIEB (ex coll. Y. Miyake):

###### Paratypes.

58 exs.: 1 ♂ ‘Mt. Pedamaran / Tana Toraja / 1985. III–17 / South SULAWESI / Soma, K. leg. // Paratype: / Malaia / sulawesi / Y. MIYAKE, 1996’. 1 ex. ‘Puncak Palopo / 1989. III. 31 / Centr. CELEBES // Paratype: / Malaia / sulawesi / Y. MIYAKE, 1996’. 3 exs. ‘Palu Pulu / C. Sulawesi / II. 1989 // Paratype: / Malaia / sulawesi / Y. MIYAKE, 1996’. [[44 exs. ‘Mt. Pedamaran / S. Sulawesi (real data is C. Sulawesi) / 20. X. 1983 (real date is 20. V. 1983) / K. Soma leg. // Paratype: / Malaia / sulawesi / Y. MIYAKE, 1996’]]. [[9 exs. ‘Mt. Pedamaran / Tana Toraja / 1983. X–20 (real date is 1983. V–20) / South SULAWESI (real data is Central SULAWESI) / Soma, K. leg. // Paratype: / Malaia / sulawesi / Y. MIYAKE, 1996’]].

###### Current status.

Valid species.

##### Malaia taoi

Taxon classificationAnimaliaColeopteraRutelidae

Miyake

EDCF8BFC-B447-5C78-880E-BBF9296C0B14

Malaia taoi Miyake, 1996: 39−40.

###### Note.

The following paratypes are deposited in RIEB (ex coll. Y. Miyake):

###### Paratypes.

4 exs.: 1 ♂ ‘37 km from / Palopo C.- / Sulawesi / 9. IV. 1982 // Paratype: / Malaia / taoi / Y. MIYAKE, 1996’. 1 ♀ ‘32 km from / Palopo / Surawesi / 10. VI. 1982 // 37 km from / Palopo C.- / Sulawesi / 10. VI. 1982 // Paratype: / Malaia / taoi / Y. MIYAKE, 1996’. 1 ♂ ‘37 km from / Palopo C.- / Sulawesi / 11. IV. 1982 // Paratype: / Malaia / taoi / Y. MIYAKE, 1996’. 1 ♀ ‘32 km from / Palopo / Surawesi / 13. VI. 1982 // 37 km from / Palopo C.- / Sulawesi / 13. VI. 1982 // Paratype: / Malaia / taoi / Y. MIYAKE, 1996’.

###### Current status.

Valid species.

##### Malaia tondanoensis

Taxon classificationAnimaliaColeopteraRutelidae

Miyake

768FB042-6157-5BEC-A61B-50BC5A907CCF

Malaia tondanoensis Miyake, 1996: 42−43.

###### Note.

The following paratype is deposited in RIEB (ex coll. Y. Miyake):

###### Paratype.

4exs.: ‘Tondano / N. Sulawesi / 10–IV–1989 / Y. Miyake leg. // Paratype: / Malaia / tondanoensis / Y. MIYAKE, 1996’.

[[1 ex. ‘Tangkoko N. / N. Sulawesi / 10–III–1989 (the date given in the original description is 10–IV–1989) / Y. Miyake leg. // Paratype: / Malaia / tondanoensis / Y. MIYAKE, 1996’]]. [[2 exs. ‘Tondano / N. Sulawesi / 9–IV–1989 (the date given in the original description is 10–IV–1989) / Y. Miyake leg. // Paratype: / Malaia / tondanoensis / Y. MIYAKE, 1996’]].

###### Current status.

Valid species.

##### Malaia toraja

Taxon classificationAnimaliaColeopteraRutelidae

Miyake

788C21F5-7F76-51EC-96D9-E8C359BBC754

Figure 2A

Malaia toraja Miyake, 2000: 110−111.

###### Note.

The holotype and following paratypes are deposited in RIEB (ex coll. Y. Miyake):

###### Holotype

**Figure 2.**
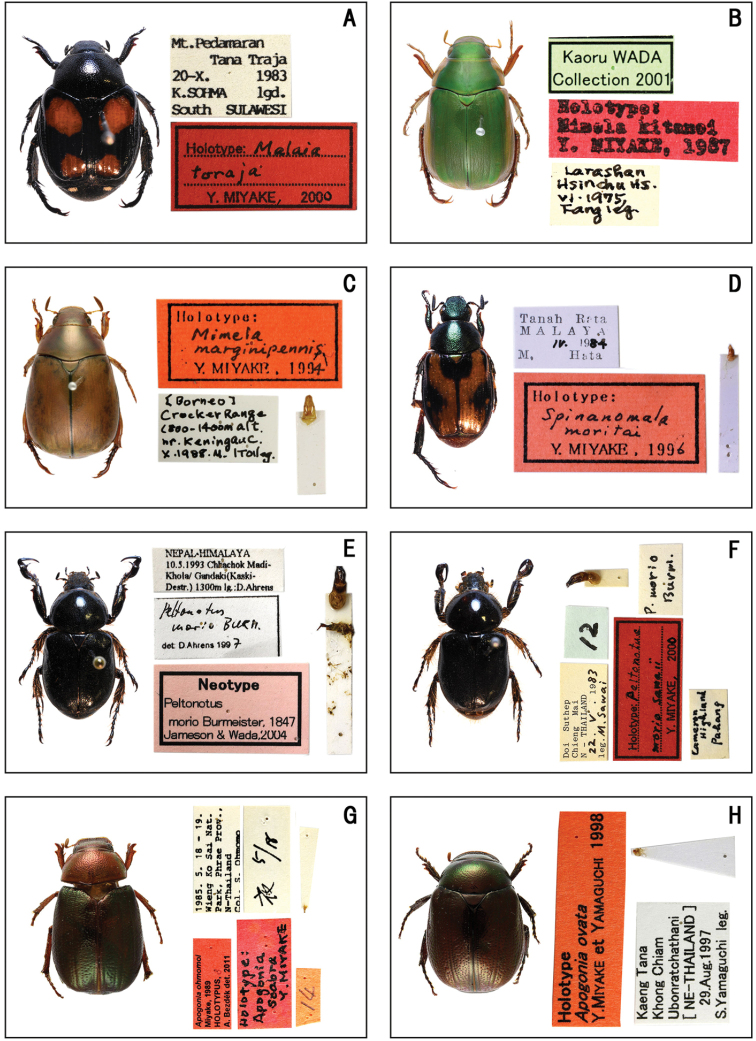
Habitus of holotype and neotype specimens. **A***Malaia toraja* Miyake **B***Mimela kitanoi* Miyake **C***Mimela marginipennis* Miyake **D***Spinanomala moritai* Miyake **E***Peltonotus morio* Burmeister **F***Peltonotus morio sawaii* Miyake **G***Apogonia ohmomoi* Miyake **H***Apogonia ovata* Miyake et Yamaguchi.

**(♂).** ‘Mt. Pedamaran / Tana Traja / 20–X. 1983 / K. SOHMA lgd. / South SULAWESI // Holotype: Malaia / toraja / Y. MIYAKE, 2000’. (Fig. 2A)

###### Paratypes.

27 exs.: 1 ♂ ‘Mt. Pedamaran / Tana Traja / 20–X. 1983 / K. SOHMA lgd. // Paratype: Malaia / toraja / Y. MIYAKE, 2000’. 9 exs. ‘Mt. Pedamaran / Tana Traja / 20–X. 1983 / K. SOHMA lgd. // Paratype: Malaia / toraja / Y. MIYAKE, 2000’. 2 exs. ‘Mt. Pedamaran / S. Sulawesi / 20. X. 1983 / K. Soma leg. // Paratype: Malaia / toraja / Y. MIYAKE, 2000’.8 exs. ‘Puncak, Palopo / VI–VII, 1989 / Central SULAWESI // Paratype: Malaia / toraja / Y. MIYAKE, 2000’. 2 exs. ‘Central SULAWESI / Puncak, Palopo / VI–VII, 1989 // Paratype: Malaia / toraja / Y. MIYAKE, 2000’. 1 ♂ ‘Puncak Palopo / 1989. / Centr. CELEBES // SI [yellow label] // Paratype: Malaia / toraja / Y. MIYAKE, 2000’. 4 exs. ‘Puncak Palopo / 1989. / Centr. CELEBES // SI [yellow label] // Paratype: Malaia / toraja / Y. MIYAKE, 2000’.

###### Current status.

Valid species.

###### Remark.

The habitus photo in the original description belongs to the female paratype with the label ‘Mt. Pedamaran / Tana Toraja / S. Sulawesi / 7, VIII, 1988 / K. Soma leg.’.

In addition to the paratypes listed above, the following specimens labeled as the paratypes are not designated in the original description: 1 ♀ ‘Mt. Pedamaran / Tana Toraja / S. Sulawesi / 7, VIII, 1988 / K. Soma leg. // SI [yellow label] // Paratype: Malaia / toraja / Y. MIYAKE, 2000’. 1 ex. ‘(Pulu Pulu) / C. Sulawesi / INDONESIA / Dec. 1988 // [yellow label] // Paratype: Malaia / toraja / Y. MIYAKE, 2000’. 2 exs. ‘Puncak, Palopo / Centr. CELEBES / 1989. I / INDONESIA // [yellow label] // Paratype: Malaia / toraja / Y. MIYAKE, 2000’. 1 ex. ‘Puncak Palopo / 1989. III. 31 / Centr. CELEBES // Paratype: Malaia / toraja / Y. MIYAKE, 2000’. 3 exs. ‘Palu, Palolo / VII 1989 / Central SULAWESI // Paratype: Malaia / toraja / Y. MIYAKE, 2000’.

#### Genus *Mimela*

##### Mimela kitanoi

Taxon classificationAnimaliaColeopteraRutelidae

Miyake

BD497983-A9A9-533E-A178-6531EAFC9751

Figure 2B

Mimela kitanoi Miyake, 1987: 7−8.

###### Note.

The holotype is deposited in RIEB (ex coll. K. Wada):

###### Holotype

**(♀).** ‘Larashan / Hsinchu Hs. / VI. 1975, / Fang leg // Holotype: / Mimela kitanoi / Y. MIYAKE, 1987 // Kaoru WADA / Collection 2001’. (Fig. 2B)

###### Current status.

Junior subjective synonym of *Mimela flavocincta* Lin, 1966, see Zorn and Bezděk (2016).

##### Mimela marginipennis

Taxon classificationAnimaliaColeopteraRutelidae

Miyake

16DCDA55-40C3-593D-96D1-6DF7CA6F3756

Figure 2C

Mimela marginipennis Miyake, 1994: 151.

###### Note.

The holotype is deposited in RIEB (ex coll. Y. Miyake):

###### Holotype

**(♂).** ‘[Borneo] / Crocker Range / 1800−1400m alt. / nr. Keningau C. / X. 1988. M. ITOI leg. // Holotype: / Mimela / marginipennis / Y. MIYAKE, 1994’. (Fig. 2C)

###### Type condition.

The aedeagus of the holotype is pinned separately, and the left mesotarsus is missing.

###### Current status.

Junior subjective synonym of *Mimela margarita* Arrow, 1910, see Wada (2001).

##### Mimela sawaii

Taxon classificationAnimaliaColeopteraRutelidae

Miyake

BD07E97C-6368-5B68-9294-1E00504CA31F

Mimela sawaii Miyake, 1994: 149−150.

###### Note.

The following paratype is deposited in RIEB (ex coll. Y. Miyake):

###### Paratype.

1 ex.: 1 ♂ ‘Crocker Range / (1000–1400 m) / Nr. Keningau C. / N. Borneo, – / 15–V–1989 // [blue label] // Paratype: / Mimela / sawaii / Y. MIYAKE, 1994’.

###### Current status.

Junior subjective synonym of *Mimela pallidicauda* Arrow, 1910, see Wada (2001).

##### Mimela suspecta

Taxon classificationAnimaliaColeopteraRutelidae

Miyake

4DBBC7A8-EAF4-5A58-9AB7-D6790A0E5BD7

Mimela suspecta Miyake, 1994: 150.

###### Note.

The following paratypes are deposited in RIEB (ex coll. Y. Miyake):

###### Paratype.

2ex.: 1 ♂ ‘Mt. Trus Madi / (SW Slopa 1200m alt.) / Sabah, MALAYSIA / 14–IV–1993 / Minoru SAWAI leg. // Paratype: / Mimela / suspecta / Y. MIYAKE, 1994’. [[1 ex. ‘Mt. Trus Madi / (SW Slopa 1200m alt.) / Sabah, MALAYSIA / 18–IV–1993 (the date given in the original description is 4–IV–1993) / Minoru SAWAI leg. // [yellow label] // Paratype: / Mimela / suspecta / Y. MIYAKE, 1994’]].

###### Current status.

Valid species.

###### Remark.

In addition to the paratypes mentioned above, the following specimen labeled as paratype is not designated in the original description: 1 ♂ ‘Trus Madi Mts. / near Keningau / Saba MALAYSIA / 1993. iv. 17–30. // F //Paratype: / Mimela / suspecta / Y. MIYAKE, 1994’.

#### Genus *Popillia*

##### Popillia iwasei

Taxon classificationAnimaliaColeopteraRutelidae

Miyake

96642894-B568-5A84-BDC0-4193ADE24E3C

Popillia iwasei Miyake, 1996: 43−44.

###### Note.

The following paratypes are deposited in RIEB (ex coll. Y. Miyake):

###### Paratypes.

6 exs.: 2 ♀ ‘Puncak, Palopo / Centr. CELEBES / 1989. I / INDONESIA // Paratype: / Popillia / iwasei / Y. MIYAKE, 1996’. 2 ♀ ‘Puncak Palopo / C. Sulawesi / 1. 1989 // Paratype: / Popillia / iwasei / Y. MIYAKE, 1996’. 1 ♀ ‘Puncak Palopo / C. Sulawesi / 1989. V / INDONESIA // SI [yellow label] // Paratype: / Popillia / iwasei / Y. MIYAKE, 1996’. [[1 ♂ ‘Palolo (the locality given in the original description is Palopo) / II. 1991 (the date given in the original description and Telnov and Zorn (2019) are I. 1989) // Paratype: / Popillia / iwasei / Y. MIYAKE, 1996’]].

###### Current status.

Valid species.

#### Genus *Spilopopillia*

##### Spilopopillia rugosa

Taxon classificationAnimaliaColeopteraRutelidae

Miyake

5E5C8BEF-A9FA-55CA-8CDE-92960A75C283

Spilopopillia rugosa Miyake, 1989a: 180−181.

###### Note.

The following paratypes are deposited in RIEB (ex coll. Y. Miyake):

###### Paratypes.

18 exs.: [[1 ex. ‘ [Malaya] / Fraser’s Hill / 29–III–1977 (the date given in the original description is 28. III. 1977) / Y. Miyake leg. // Paratype: / Spilopopillia / rugosa / Y. MIYAKE, 1989’]]. [[1 ex. ‘ [Malaya] / Freaser’s Hill (the locality given in the original description is Fraser’s Hill) / 30–III–1977 (the date given in the original description is 28. III. 1977) / Y. Miyake leg. // Paratype: / Spilopopillia / rugosa / Y. MIYAKE, 1989’]]. 6 exs. ‘ [Malaya] / Tanah Rata / 30–III–1974 / Y, Miyake leg // Paratype: / Spilopopillia / rugosa / Y. MIYAKE, 1989’. 9 exs. ‘TANARATA / MALAYA / 30 III 1974 / Y. Miyake leg. // Paratype: / Spilopopillia / rugosa / Y. MIYAKE, 1989’. [[1 ex. ‘Gentig / Hiland (the locality given in the original description is Genting Highland) MALAYA / 5. IV. 1974 (the date given in the original description is 5. IV. 1975) / Y Miyake leg. // Paratype: / Spilopopillia / rugosa / Y. MIYAKE, 1989’]].

###### Current status.

Valid species.

###### Remark.

In addition to the paratypes mentioned above, the following specimens labeled as paratypes are not designated in the original description: 8 exs. ‘Tanah Rata / MALAYA / 1. III. 1977 / Y. Miyake // Paratype: / Spilopopillia / rugosa / Y. MIYAKE, 1989’. 5 exs. ‘TANARATA / MALAYA / 28, 29, 30 III 1974 / Y. Miyake leg. // Paratype: / Spilopopillia / rugosa / Y. MIYAKE, 1989’. 1 ex. ‘84. IV // Paratype: / Spilopopillia / rugosa / Y. MIYAKE, 1989’. 2 exs. ‘Paratype: / Spilopopillia / rugosa / Y. MIYAKE, 1989’.

#### Genus *Spinanomala*

##### Spinanomala moritai

Taxon classificationAnimaliaColeopteraRutelidae

Miyake

7D0865EF-5AB8-5182-A8D6-36332D5BEA2A

Figure 2D

Spinanomala moritai Miyake, 1996: 38.

###### Note.

The holotype and following paratypes are deposited in RIEB (ex coll. Y. Miyake):

###### Holotype

**(♂).** ‘Tanah Rata / MALAYA / IV. 1984 / M. Hata // Holotype: / Spinanomala / moritai / Y. MIYAKE, 1996’. (Fig. 2D)

###### Paratypes.

11 exs.: 1 ex. ‘Tanah Rata / MALAYA / I–II. 1973 / MORITA, Kozo // Paratype: Spinanomala / moritai / Y. MIYAKE, 1996’. 2 ♂, 3 ♀ ‘Tanah Rata / MALAYA / 1. III. 1977 / Y. Miyake // Paratype: Spinanomala / moritai / Y. MIYAKE, 1996’. 3 ♂, 2 ♀ ‘Tanah Rata / MALAYA / IV. 1984 / M. Hata // Paratype: Spinanomala / moritai / Y. MIYAKE, 1996’.

###### Type condition.

The aedeagus of the holotype is pinned separately and the right metaleg is missing.

###### Current status.

Valid species.

###### Remark.

The collecting data of the specimen labelled as the holotype do not match the original description. There are two male paratypes (‘Tanah Rata / MALAYA / 1. III. 1977 / Y. Miyake // Paratype: Spinanomala / moritai / Y. MIYAKE, 1996’) that match the holotype data.

In addition to the paratypes mentioned above, the following specimen labeled as the paratype is not designated in the original description: 1 ex. ‘フレーザーヒル= furêzâhiru / MALAYA / 4 – 6. IV. 1977 / M. Hata // Paratype: Spinanomala / moritai / Y. MIYAKE, 1996’.

#### Subfamily Dynastinae

**Genus *Papuana***

##### Papuana timorensis

Taxon classificationAnimaliaColeopteraDynastidae

Miyake & Yamaya

82876068-C811-5498-A2F0-01E96156A7AF

Papuana timorensis Miyake & Yamaya, 1999: 103−104.

###### Note.

The following paratype is deposited in RIEB (ex coll. Y. Miyake):

###### Paratype.

1 ex.: 1 ♂ ‘Timor Is. / Indonesia / V. 1993 / Native Coll. // Timor / Is. [aedeagus mount] // Paratype: / Papuana / timorensis YAMAYA / Y. MIYAKE, 1999’.

###### Current status.

Valid species.

#### Genus *Peltonotus*

##### Peltonotus morio

Taxon classificationAnimaliaColeopteraDynastidae

Burmeister

AB63F9A4-2EC0-516F-B5D8-0A53585422F7

Figure 2E

Peltonotus morio Burmeister, 1847: 75.

###### Note.

The neotype is deposited in RIEB (ex coll. K. Wada):

###### Neotype

**(♂).** ‘NEPAL・HIMALAYA / 10. 5. 1993 Chhachok Madi– / Khola/ Gandaki (Kaski– / Destr.) 1300m lg: D. Ahrens //Peltonotus / morio Burn. / det: D. Ahrens 1997 // Neotype / Peltonotus / morio Burmeister, 1847 / Jameson and Wada, 2004’. (Fig. 2E)

###### Type condition.

The aedeagus of the neotype is pinned separately.

###### Current status.

Valid species.

###### Remark.

The neotype was designated by Jameson and Wada (2004).

##### Peltonotus morio sawaii

Taxon classificationAnimaliaColeopteraDynastidae

Miyake

8A162BF8-C97D-56C7-A6DB-BAC71CAA3502

Figure 2F

Peltonotus morio sawaii Miyake, 2000: 112−113.

###### Note.

The holotype and the following paratypes are deposited in RIEB (ex coll. Y. Miyake):

###### Holotype

**(♂).** ‘Doi Suthep / Chieng Mai / N–THAILAND/ 22. V. 1983 / leg. M. Sawai // 12 // P. morio / Burmi. [white label, front] // Cameron / Highland / Pahang [white label, back] // Holotype: Peltonotus / moriosawaii / Y. MIYAKE, 2000’. (Fig. 2F)

###### Paratypes.

4 exs.: 2 ♂, 1 ♀ ‘Doi Suthep / Chieng Mai / N–THAILAND / 22. V. 1983 / leg. M. Sawai // Paratype: Peltonotus / moriosawaii / Y. MIYAKE, 2000 // P. morio /Burmeister’. 1 ♀ ‘Doi Suthep / Chieng Mai / N–THAILAND / 22. V. 1983 / leg. M. Sawai // Paratype: Peltonotus / moriosawaii / Y. MIYAKE, 2000’.

###### Type condition.

The aedeagus of the holotype is pinned separately.

###### Current status.

Junior subjective synonym of *Peltonotus morio* Burmeister, 1847, see Krell and Bezděk (2016).

##### Peltonotus podocrassus

Taxon classificationAnimaliaColeopteraDynastidae

Jameson & Wada

904A01CA-2B9F-5120-B322-450EBD040D07

Peltonotus podocrassus Jameson & Wada, 2004: 34−37.

###### Note.

The following paratypes are deposited in RIEB (ex coll. Y. Miyake):

###### Paratypes.

7 exs.: 2 exs. ‘ [Malaya] / Tanah Rata / 30–III–1976 / Y. Miyake leg. // Paratype: Peltonotus peninsularis / Y. MIYAKE, 2000 // Paratype / Peltonotus podocrassus / Jameson and Wada, 2004’. 1 ♂ ‘39 miles from / Tapah, Malaya / 30–III–1976 / Y. Miyake leg. // Paratype: Peltonotus peninsularis / Y. MIYAKE, 2000 // Paratype / Peltonotus podocrassus / Jameson and Wada, 2004’. 1 ♂ ‘TANARATA / MALAYA / 30. III. 1976 / Y. Miyake leg. // Paratype: Peltonotus peninsularis / Y. MIYAKE, 2000 // Paratype / Peltonotus podocrassus / Jameson and Wada, 2004’. 1 ♂ ‘V. R〔C. H.〕/ Malaya / 21. III. 1978 // 46 // Paratype: Peltonotus peninsularis / Y. MIYAKE, 2000 // Paratype / Peltonotus podocrassus / Jameson and Wada, 2004’. 1 ex. [Malaya] / Tanah Rata / 1–IV–1984 / Y. Miyake leg. // Paratype: Peltonotus peninsularis / Y. MIYAKE, 2000 // Paratype / Peltonotus podocrassus / Jameson and Wada, 2004’. 1 ex. ‘TANARATA / MALAYA / 1. IV. 1984 / Y. Miyake // Paratype: Peltonotus peninsularis / Y. MIYAKE, 2000 // Paratype / Peltonotus podocrassus / Jameson and Wada, 2004’.

###### Current status.

Valid species.

##### Peltonotus sakaii

Taxon classificationAnimaliaColeopteraDynastidae

Miyake & Yamaya

AC24DDB8-DCB4-55C4-8285-056BEC4895A4

Peltonotus sakaii Miyake & Yamaya, 1994: 39−40, 42.

###### Note.

The following paratype is deposited in RIEB (ex coll. Y. Miyake):

###### Paratype.

1 ex.: ‘Mt. Serapi / SARAWAKU / 1991/III. // 20 // Paratype: / Peltonotus sakaii / Y. MIYAKE et YAMAYA, 1994 // Peltonotus / similis Arrow, 1931 / det. Kaoru Wada, 2004’.

###### Current status.

Junior subjective synonym of *Peltonotus similis* Arrow, 1931, see Krajcik (2012).

###### Remark.

In addition to the paratypes mentioned above, the following specimens labeled as paratypes are not designated in the original description: 1 ♂ ‘Mt. Serapi / Kuching, Sarawak / IX. 1990 / BORNEO // Paratype: / Peltonotus sakaii / Y. MIYAKE et YAMAYA, 1994 // Peltonotus / similis Arrow, 1931 / det. Kaoru Wada, 2004’. 1 ex. ‘Lembah Anai / W. Sumatra / VI. 1990 / INDONESIA // [yellow label] // Paratype: / Peltonotus sakaii / Y. MIYAKE et YAMAYA, 1994 // Peltonotus / similis Arrow, 1931 / det. Kaoru Wada, 2004’. 1 ♂ ‘Kimanis Road / Near Keningau / N. Borneo / 3–V–1994 // Paratype: / Peltonotus sakaii / Y. MIYAKE et YAMAYA, 1994 // Peltonotus / similis Arrow, 1931 / det. Kaoru Wada, 2004’. 2 exs. ‘Kimanis Road / Near Keningau / N. Borneo / 3–V–1994 // Paratype: / Peltonotus sakaii / Y. MIYAKE et YAMAYA, 1994 // Peltonotus / similis Arrow, 1931 / det. Kaoru Wada, 2004’.

##### Peltonotus sulawesiensis

Taxon classificationAnimaliaColeopteraDynastidae

Wada

119EB035-A2BA-579E-A9CF-52B909ADDDC9

Peltonotus sulawesiensis Wada, 1990: 1−2.

###### Note.

The following paratypes are deposited in RIEB (ex coll. Y. Miyake):

###### Paratypes.

4 exs.: ‘Tondano N. Celebes / INDONESIA / V 1988 / leg. N. Nisikawa // PARATYPE / Peltonotus sulawesiensis / Kaoru WADA, 1990’.

###### Current status.

Junior subjective synonym of *Neohyphus celebesus* Heller, 1896, see Kuijten (1994).

#### Subfamily Melolonthinae

**Genus *Amiserica***

##### Amiserica rufidula

Taxon classificationAnimaliaColeopteraMelolonthidae

Nomura

A33D6B93-3142-5882-A021-B0E4A64F3B43

Amiserica rufidula Nomura, 1974: 84.

###### Note.

The following paratypes are deposited in RIEB (ex coll. S. Nomura):

###### Paratypes.

2 exs.: 1 ♂ ‘Li-shan / Taiwan / 29. VII. 1974 / Y. Miyake // ♂ // PARATYPE / Amiserica / rufidula / NOMURA (1974)’. 1 ♂ ‘Li-shan / Taiwan / 1. VIII. 1974 / Y. Miyake // ♂ // PARATYPE / Amiserica / rufidula / NOMURA (1974)’.

###### Current status.

Valid species.

#### Genus *Apogonia*

##### Apogonia fujiokai

Taxon classificationAnimaliaColeopteraMelolonthidae

Miyake & Yamaya

E96EFD87-9EE6-5DA3-BFF4-D582BBE73B5B

Apogonia fujiokai Miyake & Yamaya, 1997: 6−7.

###### Note.

The following paratype is deposited in RIEB (ex coll. Y. Miyake):

###### Paratype.

1 ex.: ‘Flores Is. / Indonesia / May. 1995 // Y // Paratype: / Apogonia fujiokai / Y. MIYAKE et YAMAYA, 1997’.

###### Current status.

Valid species.

##### Apogonia fusciventris

Taxon classificationAnimaliaColeopteraMelolonthidae

Miyake & Yamaya

C146E0CC-97AF-540E-852D-EEFA391B54EC

Apogonia fusciventris Miyake & Yamaya, 1997: 5−6.

###### Note.

The following paratype is deposited in RIEB (ex coll. Y. Miyake):

###### Paratype.

1 ex.: ‘Vangvieng / P. Vietiane / LAO. P. D. R. / 25–26. MAY. 1994. / K. Miura–leg. // Y // Paratype: Apogonia / fuscoventris / Y. MIYAKE et YAMAYA, 1997’.

###### Current status.

Junior subjective synonym of of *Apogonia striatipennis* Frey, 1971, see Kobayashi (2010).

###### Remark.

The species name on the paratype label is ‘*fuscoventris*’, which is invalid. Probably, the name was confused with *fusciventris* by Y. Miyake.

##### Apogonia hongkongica

Taxon classificationAnimaliaColeopteraMelolonthidae

Miyake

05D72C6E-19CC-5739-B9EA-0DFAAD654538

Apogonia hongkongica Miyake, 1989b: 38−39.

###### Note.

The following paratypes are deposited in RIEB (ex coll. Y. Miyake):

###### Paratypes.

11 exs.: 1 ♂ ‘Hong Kong / China / C. K. Yu / 1974. V. [white label, front] // Native Collector / legt. [white label, back] // Paratype: / Apogonia / hongkongica / Y. MIYAKE, 1989’. 1 ♂ ‘Hong Kong / China / V. 1974 / C. K. Yu [white label, front] // Legt. / Native / Collector [white label, back] // Paratype: / Apogonia / hongkongica / Y. MIYAKE, 1989’. 1 ♂ ‘Hong Kong / China / V. 1974 / C. K. Yu [white label, front] // Native Collector [white label, back] // Paratype: / Apogonia / hongkongica / Y. MIYAKE, 1989’. 1 ♀ ‘♀ // Hong Kong / China / V. 1974 / C. K. Yu [white label, front] // Legt. / Native / Collector // Paratype: / Apogonia / hongkongica / Y. MIYAKE, 1989’. 1 ♀ ‘♀ // Hong Kong / China / V. 1974 / C. K. Yu [white label, front] // Native / Collector / legt. // Paratype: / Apogonia / hongkongica / Y. MIYAKE, 1989’. 1 ♀ ‘Hong Kong / 1974 / From C. K. Yu ♀ // Paratype: / Apogonia / hongkongica / Y. MIYAKE, 1989’. 1 ex. ‘Hong Kong / China / V. 1974 / C. K. Yu [white label, front] // Legt. / native / Collector [white label, back] // Paratype: / Apogonia / hongkongica / Y. MIYAKE, 1989’. 2 exs. ‘Hang Kong / China / V. 1974 / C. K. Yu Col [white label, front] // Native / Collector / legt. // Paratype: / Apogonia / hongkongica / Y. MIYAKE, 1989’. 2 exs. ‘Hong Kong / V. 1974 / From C. K. Yu // Paratype: / Apogonia / hongkongica / Y. MIYAKE, 1989’.

###### Current status.

Valid species.

###### Remark.

In addition to the paratypes mentioned above, the following specimens labeled as paratypes are not designated in the original description: 1ex. ‘Hong Kong / 1975 / From. C. K-Yu // Paratype: Apogonia / hongkongica / Y. MIYAKE, 1989’. 1 ♀ ‘Hong Kong / ♀ 1975 [white label, front] // from / C. K. Yu [white label, back] // Paratype: / Apogonia / hongkongica / Y. MIYAKE, 1989’.

##### Apogonia inconstans

Taxon classificationAnimaliaColeopteraMelolonthidae

Kobayashi & Bezděk

9D598169-F5EA-5D40-BFB9-2D9F962506CA

Apogonia inconstans Kobayashi & Bezděk, 2011: 64−65.

###### Note.

The following paratypes are deposited in RIEB (ex coll. A. Bezděk):

###### Paratypes.

4 exs.: 1 ♂ ‘1985. 5. 18−19. / Wieng Ko Sai Nat. / Park, Phrae Prov., / N-Thailand / Col. S. Ohmomo [white label, front] // 5/18 夜= yoru [white label, back] // PARATYPE / *Apogonia* / *inconstans* / Kobayashi et Bezděk, 2011’. 1 ex. ‘1985. 5. 18−19. / Wieng Ko Sai Nat. / Park, Phrae Prov., / N-Thailand / Col. S. Ohmomo [white label, front] // 5/18 夜= yoru [white label, back] // PARATYPE / *Apogonia* / *inconstans* / Kobayashi et Bezděk, 2011’. 1 ex. ‘1985. 5. 18−19. / Wieng Ko Sai Nat. / Park, Phrae Prov., / N-Thailand / Col. S. Ohmomo [white label, front] // 夜= yoru 5/18 [white label, back] // PARATYPE / *Apogonia* / *inconstans* / Kobayashi et Bezděk, 2011’. 1 ex. ‘1985. 5. 18−19. / Wieng Ko Sai Nat. / Park, Phrae Prov., / N-Thailand / Col. S. Ohmomo [white label, front] // night [white label, back] // PARATYPE / *Apogonia* / *inconstans* / Kobayashi et Bezděk, 2011’.

###### Current status.

Valid species.

##### Apogonia ohmomoi

Taxon classificationAnimaliaColeopteraMelolonthidae

Miyake

61714DF5-6C92-52D4-BA22-3816312E7F35

Figure 2G

Apogonia ohmomoi Miyake, 1989: 177−178.

###### Note.

The holotype is deposited in RIEB (ex coll. Y. Miyake):

###### Holotype

**(♂).** ‘1985. 5. 18−19. / Wieng Ko Sai Nat. / Park, Phrae Prov., / N−Thailand / Col. S. Ohmomo [white label, front] // 夜= yoru 5/18 [white label, back] // 14 // Holotype: / Apogonia / scabra / Y. MIYAKE // *Apogonia ohmomoi* / Miyake, 1989 / HOLOTYPUS, ♂ / A. Bezděk det. 2011’. (Fig. 2G)

###### Type condition.

The aedeagus of the holotype is pinned separately, and the right antenna, right and left protarsus, right mesoclaw, left mesotarsus, right metatarsus and mesonotum are missing.

###### Current status.

Valid species.

###### Remark.

The label ‘*Apogonia scabra*’ was apparently accidentally put by Miyake.

##### Apogonia ovata

Taxon classificationAnimaliaColeopteraMelolonthidae

Miyake & Yamaguchi

3A00381F-AB5C-57B8-AF75-93F23E810DEA

Figure 2H

Apogonia ovata Miyake & Yamaguchi, 1998: 25−27.

###### Note.

The holotype is deposited in RIEB (ex coll. Y. Miyake):

###### Holotype

**(♂).** ‘Kaeng Tana / Khong Chiam / Ubonratchathani / [NE-THAILAND] / 29. Aug. 1997 / S. Yamaguchi leg. // Holotype / *Apogonia ovata* / Y. MIYAKE et YAMAGUCHI 1998’. (Fig. 2H)

###### Type condition.

The aedeagus of the holotype is pinned separately.

###### Current status.

Senior synonym of *Apogonia miyakei* Bezděk, 2004, see Bezděk (2004).

##### Apogonia terminalis

Taxon classificationAnimaliaColeopteraMelolonthidae

Miyake, Yamaguchi & Akiyama

788E5484-1931-57BD-9F86-4438A8A1B71B

Figure 3A

Apogonia terminalis Miyake, Yamaguchi & Akiyama, 2002: 56−58.

###### Note.

The holotype and following paratypes are deposited in RIEB (ex coll. Y. Miyake):

###### Holotype

**Figure 3.**
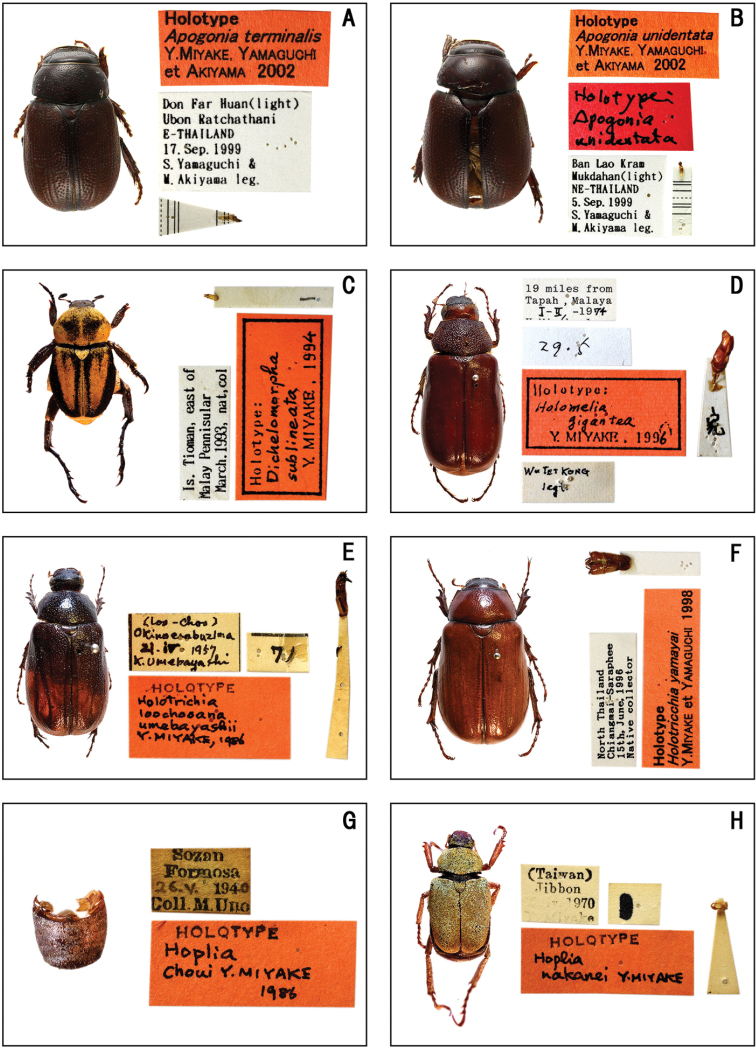
Habitus of holotype specimens. **A***Apogonia terminalis* Miyake, Yamagushi et Akiyama **B***Apogonia unidentata* Miyake, Yamaguchi et Akiyama **C***Dichelomorpha sublineata* Miyake **D***Holomelia gigantea* Miyake **E***Holotrichia loochooana umebayashii* Miyake **F***Holotrichia yamayai* Miyake et Yamaguchi **G***Hoplia choui* Miyake **H***Hoplia nakanei* Miyake.

**(♂).** ‘Don Far Huan (light) / Ubon Ratchathani / E-THAILAND / 17. Sep. 1999 / S. Yamaguchi & / M. Akiyama leg. // Holotype / *Apogonia terminalis* / Y. MIYAKE, YAMAGUCHI / et AKIYAMA 2002’. (Fig. 3A)

###### Paratypes.

4exs.: 1 ♂ ‘Ban Lao Kram / Mukdahan (light) / NE-THAILAND / 5. Sep. 1999 / S. Yamaguchi & / M. Akiyama leg. // Paratype / *Apogonia terminalis* / Y. MIYAKE, YAMAGUCHI / et AKIYAMA 2002’. 3 ♀ ‘Don Far Huan (light) / Ubon Ratchathani / E-TAILAND / 17. Sep. 1999 / S. Yamaguchi & / M. Akiyama leg. // ♀ // Paratype / *Apogonia terminalis* / Y. MIYAKE, YAMAGUCHI / et AKIYAMA 2002’.

###### Type condition.

The aedeagus of the holotype is pinned separately, and the left proclaw, right and left mesotarsus, right metaclaw and left metatarsus are missing.

###### Current status.

Valid species.

##### Apogonia unidentata

Taxon classificationAnimaliaColeopteraMelolonthidae

Miyake, Yamaguchi & Akiyama

A81AAD25-5775-590C-89DB-534062211D3B

Figure 3B

Apogonia unidentata Miyake, Yamaguchi & Akiyama, 2002: 55−56.

###### Note.

The holotype and following paratypes are deposited in RIEB (ex coll. Miyake):

###### Holotype

**(♂).** ‘Ban Lao Kram / Mukdahan (light) / NE-THAILAND / 5. Sep. 1999 / S. Yamaguchi & / M. Akiyama leg. // Holotype / Apogonia / unidentata // Holotype / *Apogonia unidentata* / Y. MIYAKE, YAMAGUCHI / et AKIYAMA 2002’. (Fig. 3B)

###### Paratypes.

6 exs.: 4exs. ‘Ban Lao Kram / Mukudahan (light) / NE-THAILAND / 5. Sep. 1999 / S. Yamaguchi & / M. Akiyama leg. // Paratype / *Apogonia unidentata* / Y. MIYAKE, YAMAGUCHI / et AKIYAMA 2002’. 1 ex. ‘Ban Lao Kram / Mukudahan (light) / NE-THAILAND / 5. Sep. 1999 / S. Yamaguchi & / M. Akiyama leg. // 63 // Paratype / *Apogonia unidentata* / Y. MIYAKE, YAMAGUCHI / et AKIYAMA 2002’. 1 ex. ‘Ban Lao Kram / Mukudahan (light) / NE-THAILAND / 14. Sep. 1999 / S. Yamaguchi & / M. Akiyama leg. / シミレア= shimirea // Paratype / *Apogonia unidentata* / Y. MIYAKE, YAMAGUCHI / et AKIYAMA 2002’.

###### Type condition.

The aedeagus of the holotype is pinned separately, and the right protarsus and right and left metatarsi are missing.

###### Current status.

Valid species.

#### Genus *Dichelomorpha*

##### Dichelomorpha sublineata

Taxon classificationAnimaliaColeopteraMelolonthidae

Miyake

A53A781B-F26E-59A5-B014-70E43FD6E0FD

Figure 3C

Dichelomorpha sublineata Miyake, 1994: 140−141.

###### Note.

The holotype and following paratypes are deposited in RIEB (ex coll. Y. Miyake):

###### Holotype

**(♂).** ‘Is. Tioman, east of / Malay Pennisular March. 1993, nat, col // 1 [aedeagus mount] // Holotype: / Dichelomorpha / sublineata / Y. MIYAKE, 1994’. (Fig. 3C)

###### Paratypes.

34 exs.: 10 ♂ ‘Is. Tioman, east of / Malay Pennisular / March. 1993. nat, col // Paratype: / Dichelomorpha / sublineata / Y. MIYAKE, 1994’. 14 exs. ‘Is. Tioman, east of / Malay Pennisular / March. 1993. nat, col // Paratype: / Dichelomorpha / sublineata / Y. MIYAKE, 1994’. 1 ♂ ‘Is. Tioman, east of / Malay Pennisular / March. 1993. nat, col // 11 // Paratype: / Dichelomorpha / sublineata / Y. MIYAKE, 1994’. 1 ex. ‘Is. Tioman, east of / Malay Pennisular / March. 1993. nat, col // 4 // Paratype: / Dichelomorpha / sublineata / Y. MIYAKE, 1994’. [[8 exs. ‘Tioman Is. / E. of Malaya / III–1933 (the date given in the original description is III–1993), W. // Paratype: / Dichelomorpha / sublineata / Y. MIYAKE, 1994’]].

###### Type condition.

The aedeagus of the holotype is pinned separately. The right mesotarsus is missing.

###### Current status.

Valid species.

###### Remark.

Miyake (1994) did not provide a photograph of the holotype. Apparently he provided a photograph of a paratype, but we could not trace that specimen.

#### Genus *Holomelia*

##### Holomelia gigantea

Taxon classificationAnimaliaColeopteraMelolonthidae

Miyake

57CD590E-E82D-5FDD-95EA-5949C1FA3046

Figure 3D

Holomelia gigantea Miyake, 1996: 35−36.

###### Note.

The holotype and following paratypes are deposited in RIEB (ex coll. Y. Miyake).

###### Holotype

**(♂).** ‘19 miles from / Tapah, Malaya / 1–II–1974 / Y. Miyake leg [white label, front] // Wu TET KONG / legt. [white label, back] // 29・5 // 家= ie [aedeagus mount] // Holotype: / Holomelia / gigantea / Y. MIYAKE, 1996’. (Fig. 3D)

###### Paratypes.

2 exs.: 1 ♂ ‘Cameron H. / MALAYA / Jan–Feb 1974 / Y. Miyake leg. // 30.5 // Paratype: / Holomelia gigantea / Y. MIYAKE, 1996 // 19 miles from / Tapah, Malaya / 1–II–1974 [white label, front] // Wu Tet Kong legt. [white label, back]’. 1 ex. ‘19 miles from / Tapah, Malaya / 1–II–1974 [white label, front] // Wu Tet Kong legt. [white label, back] // Paratype: / Holomelia gigantea / Y. MIYAKE, 1996’.

###### Type condition.

The aedeagus of the holotype is pinned separately.

###### Current status.

Valid species.

###### Remark.

Miyake (1996) did not provide a photograph of the holotype. Apparently he provided a photograph of a paratype, but we could not trace that specimen.

#### Genus *Holotrichia*

##### Holotrichia loochooana umebayashii

Taxon classificationAnimaliaColeopteraRhodomelaceae

Miyake

5A25A8A7-0A63-570C-A082-7BBD644E57A0

Figure 3E

Holotrichia loochooana umebayashii Miyake, 1986a: 1−2.

###### Note.

The holotype is deposited in RIEB (ex coll. Y. Miyake):

###### Holotype

**(♂).** ‘〔Loo-Choo〕/ Okinoerabuzima / 21. IV. 1957. / K. Umebayashi // 7 [white label, mounted on a part of tarsus] // HOLOTYPE / Holotrichia / loochooana / umebayashii / Y. MIYAKE, 1986’. (Fig. 3E)

###### Type condition.

The aedeagus of the holotype is pinned separately. The left protarsus and the right and left metatarsus are missing.

###### Current status.

Junior subjective synonym of *Nigrotrichia loochooana okinawana* (Nomura, 1964), see Matsumoto (2016).

###### Remark.

The collecting date in the original description is 1.V.1957, which disagrees with the label data.

##### Holotrichia yamayai

Taxon classificationAnimaliaColeopteraRhodomelaceae

Miyake & Yamaguchi

78F48E7C-5440-547E-928D-E7C3EFA269F1

Figure 3F

Holotrichia yamayai Miyake & Yamaguchi, 1998: 27−28.

###### Note.

The holotype and following paratype are deposited in RIEB (ex coll. Y. Miyake):

###### Holotype

**(♂).** ‘North Thailand / Chiangmai-Saraphee / 15th, June, 1996 / Native collector // Holotype / *Holotricchia yamayai* / Y. MIYAKE et YAMAGUCHI 1998’. (Fig. 3F)

###### Paratype.

1 ex.: 1 ♀ ‘Kaeng Tana / Khong Chiam / Ubonratohathani / [NE-THAILAND] / 27. Aug. 1997 / S. Yamaguchi leg. // Paratype / Holotrichia yamayai / Y. MIYAKE et YAMAGUCHI 1998’.

###### Type condition.

The aedeagus of the holotype is pinned separately.

###### Current status.

Valid species.

#### Genus *Hoplia*

##### Hoplia choui

Taxon classificationAnimaliaColeopteraMelolonthidae

Miyake

3894136C-383B-503D-8CEE-E5FC9E0DBCFB

Figure 3G

Hoplia choui Miyake, 1986b: 203−204.

###### Note.

The holotype is deposited in RIEB (ex coll. Y. Miyake):

###### Holotype

**(♂).** ‘Sozan / Formosa / 26. V. 1940 / Coll. M. Uno // HOLOTYPE / Hoplia / Choui Y. MIYAKE / 1986’. (Fig. 3G)

###### Type condition.

Abdomen only.

###### Current status.

Valid species.

###### Remark.

Most part of the holotype specimen was destroyed by *Anthrenus verbasci*.

##### Hoplia nakanei

Taxon classificationAnimaliaColeopteraMelolonthidae

Miyake

91A3288A-ADAE-5303-B357-F3A40BF0FAAA

Figure 3H

Hoplia nakanei Miyake, 1986b: 207.

###### Note.

The holotype and following paratypes are deposited in RIEB (ex coll. Y. Miyake):

###### Holotype

**(♂).** ‘ (Taiwan) / Jibbon / 2. iv. 1970 / Y. Miyake // 1 // HOLOTYPE / Hoplia / nakanei Y. MIYAKE’. (Fig. 3H)

###### Paratypes.

29 exs.: 4 exs. ‘ [Taiwan] / 六亀= liugui．石山= shi shan / 9–VI–1976 / Y. Miyake leg. // Paratype / Hoplia / nakanei / Y. Miyake, 1986’. 5 exs. ‘ [Taiwan] / Liukuei 石山= shi shan / 9–VI–1976 / Y. Miyake leg. // Paratype / Hoplia / nakanei / Y. Miyake, 1986’. 1 ex. ‘ [Taiwan] / 六亀= liugui．石山= shi shan / 9–VI–1976 / 陳文竜= chen wenzhao leg. // Paratype / Hoplia nakanei / Y. Miyake, 1986’. 4 exs. ‘ [Taiwan] / Baibara / –V–1963 / T. Shirozu leg. // Paratype / Hoplia / nakanei / Y. Miyake, 1986’. 1 ex. ‘ [Taiwan] / Baibara / –V–1963 / Shirozu leg. // Paratype / Hoplia / nakanei / Y. Miyake, 1986’. 1 ex. ‘ [Taiwan] / Baibara / –V–1963 / T. Shirozu // Paratype: Hoplia / nakanei Y. Miyake, 1986’. 1 ♂ ‘六亀= liugui．石山= shi shan / alt. 2000 m / 9. VI. 1976 // Paratype: / Hoplia / nakanei / Y. MIYAKE, 1986’. 1 ex. ‘FENCHIFO / FORMOSA / 24. V. 1975 / K. MATSUDA // Paratype: / Hoplia nakanei / Y. MIYAKE, 1986’. 1 ex. ‘Swakang / Taiwan / 15. VI. 1970 / Fukuda // Paratype: / Hoplia nakanei / 1986. Y. MIYAKE’. 1 ex. ‘NANSHANCHI / TAIWAN / 1. IV. 1970 / H. NOMURA // PARATYPE / Hoplia / nakanei / Y. MIYAKE, 1986’. [[4 exs. ‘ [Taiwan] / Baibara / V. 1965 (the date given in the original description is V. 1963) / T. Shirozu // Paratype / Hoplia nakanei / Y. Miyake’]]. [[2 exs. ‘ [Taiwan] / Baibara / V. 1965 (the date given in the original description is V. 1963) / T. Shirozu // Paratype / Hoplia nakanei / Y. Miyake, 1986’]]. [[1 ♂ ‘ [Taiwan] / Baibara / V. 1965 (the date given in the original description is V. 1963) / Col. T. Shirozu // Paratype / Hoplia nakanei / Y. Miyake, 1986’]]. [[1 ex. ‘ [Taiwan] / Baibara / V. 1965 (the date given in the original description is V. 1963) / Col. T. Shirozu // Paratype / Hoplia nakanei / Y. Miyake, 1986’]]. [[1 ex. ‘ [Taiwan] / Baibara / V. 1965 (the date given in the original description is V. 1963) / Col. T. Shirozu // Paratype / Hoplia nakanei / 1986. Y. Miyake’]].

###### Type condition.

The aedeagus of the holotype is pinned separately.

###### Current status.

Valid species.

###### Remark.

The label data the holotype do not match the original description. There is one male paratype (‘六亀= liugui．石山= shi shan / alt. 2000 m / 9. VI. 1976 // Paratype: / Hoplia / nakanei / Y. MIYAKE, 1986’) that agrees with the data of the holotype. Probably the type labels were mixed up and this specimen should be considered the holotype.

In addition to the paratypes listed above, the following specimens labeled as paratypes are not designated in the original description: 1 ex. ‘ [Taiwan] / Liukuei / –V–1963 / T. Shirozu col. // Paratype / Hoplia / nakanei / Y. Miyake, 1986’. 1 ex. ‘APR II. 1971 / 榮華= ronghua / B-S. CHANG // PARATYPE / Hoplia / nakanei / Y. MIYAKE, 1986’. 1 ex. ‘Paratype / Hoplia nakanei / Y. MIYAKE, 1986’.

##### Hoplia nengkaoshana

Taxon classificationAnimaliaColeopteraMelolonthidae

Miyake

C1DE97FD-1C02-5F60-8CAE-CE2832FF5B81

Hoplia nengkaoshana Miyake, 1986b: 211.

###### Note.

The allotype and following paratypes are deposited in RIEB (ex coll. Y. Miyake):

**Allotype (♀).** ‘Allotype // mt. Noko (2650 m) / V. 20–VI. 2, 1966 // 1 // PARATYPE / Hoplia / nengkaoshana / Y. MIYAKE, 1986’.

###### Paratypes.

3ex.: 1 ♂ ‘mt. Nōhō / C-Formosa / V–20 ～ Vi–21 1966 // PARATYPE / Hoplia / nengkaoshana / Y. MIYAKE, 1986’. 1 ♂ ‘MT. HÔFAN / FORMOSA / 5–V. 73 / T. OCHI // 1 // PARATYPE / Hoplia / nengkaoshana / Y. MIYAKE, 1986 // Hoplia / inornata / H. Kobayashi, 1990 / Det. H. Kobayashi, 2015’. 1 ex. ‘JUL. 16. 1966 / 合欢山= hehuan shan / B. S. Chang // PARATYPE / nengkaoshana / Y. MIYAKE, 1986 // Hoplia / inornata / H. Kobayashi, 1990 / Det. H. Kobayashi, 2015’.

###### Current status.

Valid species.

###### Remark.

In addition to the paratypes mentioned above, the following specimens labeled as paratypes are not designated in the original description: 1 ex. ‘台中= tai chung 和平郷= heping xiang 早等村= zaodeng cun / 横貫公路= hengguan gonglu. 梨山站= lishan zhan / 海抜= kaibatsu 1679–2196 m. / 13–16. V. 1958. 陶家駒= tao jiayu // PARATYPE / Hoplia / nengkaoshana / Y. MIYAKE, 1986’. 1 ex. ‘AUG 13. 1970 / 新坡= xinpo / B-S. CHANG // PARATYPE / Hoplia / nengkaoshana / Y. MIYAKE, 1986’.

##### Hoplia simillima

Taxon classificationAnimaliaColeopteraMelolonthidae

Miyake

E2A3BA86-D46A-575B-834E-1B0CF8CB43B4

Figure 4A

Hoplia simillima Miyake, 1986b: 206−207.

###### Note.

The holotype and following paratypes are deposited in RIEB (ex coll. Y. Miyake):

###### Holotype

**Figure 4.**
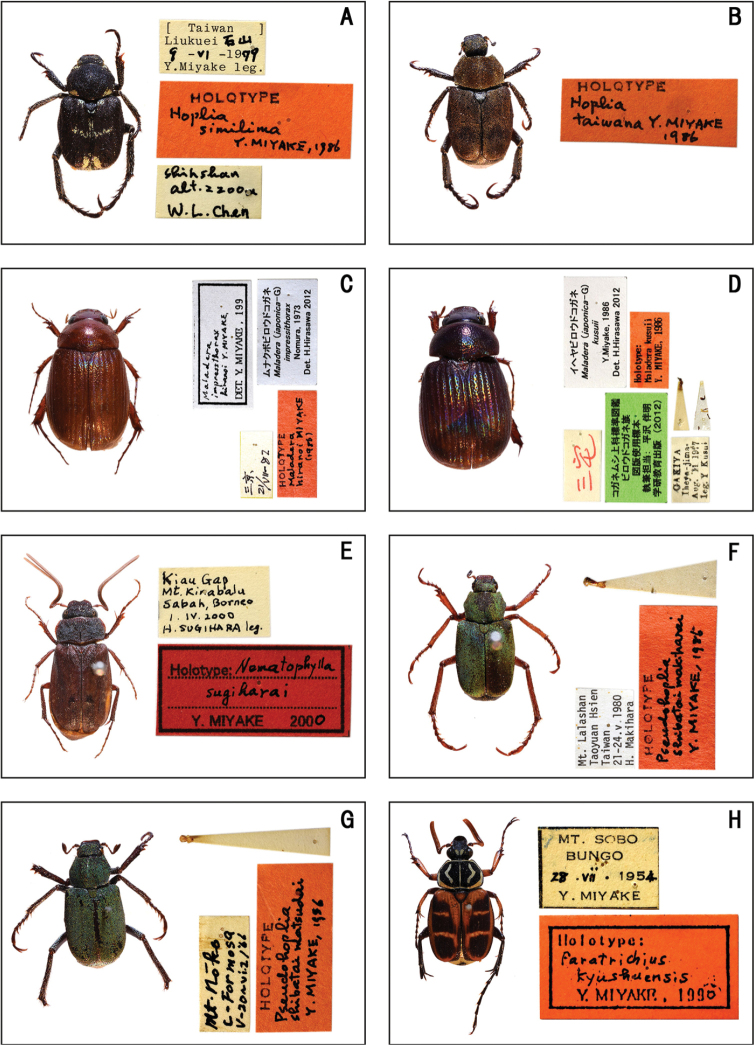
Habitus of holotype specimens. **A***Hoplia simillima* Miyake **B***Hoplia taiwana* Miyake **C***Maladera hiranoi* Miyake **D***Maladera kusuii* Miyake **E***Nematophylla sugiharai* Miyake **F***Pseudohoplia shibatai makiharai* Miyake **G***Pseudohoplia shibatai matsudai* Miyake **H***Paratrichius kyushuensis* Miyake.

**(♂).** ‘ [Taiwan] / Liukuei 石山= shi shan / 9–VI–1979 / Y. Miyake leg. // HOLOTYPE / Hoplia / simillima / Y. MIYAKE, 1986’. (Fig. 4A)

###### Paratypes.

5 exs.: 3 exs. ‘(FORMOSA) / Baibara / –V–1969 // Paratype / Hoplia / simillima / 1989, Y. Miyake’. 2 exs. ‘(FORMOSA) / Baibara / –V–1969 / T. Shirozu // Paratype / Hoplia / simillima / 1989, Y. Miyake’.

###### Current status.

Valid species.

###### Remark.

In addition to the paratypes mentioned above, the following specimens labeled as paratypes are not designated in the original description: 1 ex. ‘ [Taiwan] / Liukuei 石山= shi shan / 9–VI–1979 / Y. Miyake leg. // Paratype / Hoplia / simillima / 1989, Y. Miyake’. 1 ♂ ‘石= ishi // [Taiwan] / 六亀= liugui 石山= shi shan / 9–VI–1976 / Y. Miyake leg. // Paratype / Hoplia / simillima / 1989, Y. Miyake’. 1 ex. ‘(FORMOSA) / Mt. Ali / 14 V 1968 / Y. HAYASHI // Paratype’.

##### Hoplia taiwana

Taxon classificationAnimaliaColeopteraMelolonthidae

Miyake

8EF55CF4-A983-5C6F-BBBD-8F7CFDAF720C

Figure 4B

Hoplia taiwana Miyake, 1986b: 204.

###### Note.

The holotype and following paratypes are deposited in RIEB (ex coll. Y. Miyake):

###### Holotype

**(♂).** ‘HOLOTYPE / Hoplia / Taiwana Y. MIYAKE / 1986’. (Fig. 4B)

###### Paratypes.

9 exs.: 1 ex. ‘NANSHANCHI / TAIWAN / 3. IV. 1970 / H. NOMURA // PARATYPE / Hoplia taiwana / Y. MIYAKE, 1986’. 1 ex. ‘NANSHANCHI / TAIWAN / 4. IV. 1970 / H. NOMURA // PARATYPE / Hoplia taiwana / Y. MIYAKE, 1986’. 1 ex. ‘NANSHANCHI / TAIWAN / 5. IV. 1970 / Y. KIYOYAMA // PARATYPE / Hoplia taiwana / Y. MIYAKE, 1986’. 1 ex. ‘SUNGKANG / FORMOSA / 29. VI. 1971 / Y. MAEDA // PARATYPE / Hoplia taiwana / Y. MIYAKE, 1986’. 1 ex. ‘NANSHANCHI / TAIWAN / 31. III. 1981 / Y. YAMAMOTO // Paratype / Hoplia / taiwana / Y. MIYAKE, 1986’. 1 ex. ‘NANSHANCHI / TAIWAN / 1. IV. 1981 / F. KIMURA // Paratype / Hoplia / taiwana Y. MIYAKE / (1968)’. 1 ex. ‘NANSHANCHI / TAIWAN / 2. IV. 1981 / F. KIMURA // Paratype / Hoplia / taiwana Y. MIYAKE’. 1 ex. ‘NANSHANCHI / TAIWAN / 2. IV. 1981 / F. KIMURA // Paratype / Hoplia taiwana / 1986 Y. MIYAKE’. 1 ex. ‘NANSHANCHI / TAIWAN / 3. IV. 1981 / Y. YAMAMOTO // PARATYPE / Hoplia taiwana / Y. MIYAKE, 1986’.

###### Current status.

Valid species.

###### Remark.

The locality label data of the holotype are missing but the specimen agrees with the original description.

In addition to the paratypes mentioned above, the following specimens labeled as paratypes were not designated in the original description: 3 exs. ‘ [Taiwan] / Liukuei 石山= shi shan / 9–VI–1976 / Y. Miyake leg. // PARATYPE / Hoplia / taiwana / Y. MIYAKE, 1986’. 1 ex. ‘ [Taiwan] / 六亀= liugui 石山= shi shan / 9–VI–1976 / Y. Miyake leg. // PARATYPE / Hoplia / taiwana / Y. MIYAKE, 1986’. 2 exs. ‘Mt. YUSHAN / TAIWAN / 8. VI. 1980 / M. YAMAMOTO // Paratype / Hoplia / taiwana Y. MIYAKE’. 4 exs. ‘TAIWAN / 陽明山= mt. yangmingshan / 20. IV. 1956 / COL. K. S. LIN // Paratype / Hoplia / taiwana / Y. MIYAKE, 1986’. 1 ex. ‘LUSHAN / FORMOSA / 13–IV. 74 / S. TAKEDA // PARATYPE / Hoplia / taiwana / Y. MIYAKE, 1986’. 1 ex. ‘榮華= ronghua / APR. 7. 1971 / BS, CHANG // PARATYPE / Hoplia taiwana / Y. MIYAKE, 1986’. 1 ex. ‘ [Taiwana] / Baibara / –V–1965 / T. Shirozu leg. // Paratype: Hoplia / taiwana Y. MIYAKE. 1986’.

#### Genus *Maladera*

##### Maladera hiranoi

Taxon classificationAnimaliaColeopteraMelolonthidae

Miyake

E833BA3A-DD9C-5399-B697-9ED67E6D5A63

Figure 4C

Maladera hiranoi Miyake, 1986b: 3−5.

###### Note.

The holotype is deposited in RIEB (ex coll. Y. Miyake):

###### Holotype

**(♂).** ‘三京= mikyô / 21/VII–82 // HOLOTYPE / Maladera / hiranoi MIYAKE / (1986) // Maladera / impressthorax / hiranoi Y. MIYAKE / DET. Y. MIYAKE, 199 // ムナクボビロウドコガネ= munakubobirôdokogane / *Maladera* (*japonica*–G) / *impressithorax* / Nomura, 1973 / Det. H. Hirasawa 2012’. (Fig. 4C)

###### Current status.

Junior subjective synonym of *Maladera okinoerabuana* Kobayashi, 1978, see Ahrens and Bezděk (2016).

###### Remark.

The date on the collecting data label of the holotype is 21.VII.1982, but the collecting date quoted in the original description is 22.VII.1982. Probably, Miyake made a typographical error when writing the description.

##### Maladera kobayashii

Taxon classificationAnimaliaColeopteraMelolonthidae

Nomura

4BCAA1B3-A149-563D-A100-6D1F62049CE7

Maladera kobayashii Nomura, 1974: 106−107.

###### Note.

The following paratypes are deposited in RIEB (ex coll. S. Nomura):

###### Paratypes.

2 exs.: 1 ♂ ‘Li-shan / Taiwan / 29. VII. 1974 / Y. Miyake // ♂ // PARATYPE / Maladera (s. str) / kobayashii / NOMURA (1974)’. 1 ♀ ‘Li-shan / Taiwan / 29. VII. 1974 / Y. Miyake // ♀ // PARATYPE / Maladera / kobayashii / NOMURA (1974)’.

###### Current status.

Valid species.

##### Maladera kusuii

Taxon classificationAnimaliaColeopteraMelolonthidae

Miyake

0D97437C-AEE7-5D0A-9C7B-AD4C918AD396

Figure 4D

Maladera kusuii Miyake, 1986b: 2−3.

###### Note.

The holotype is deposited in RIEB (ex coll. Y. Miyake):

###### Holotype

**(♂).** ‘三宅= miyake / コガネムシ上科標準図鑑= koganemushijyôka-hyôjyunzukan / ビロウドコガネ族= birôdokoganezoku / 図鑑使用標本= zukanshiyôhyôhon / 執筆担当= shippitsutantô：平沢 伴明= hirasawa tomoaki / 学研教育出版= gakkenkyôikushuppan (2012) // GAKIYA / Iheya-jima / Aug. 11 1977 / leg. Y Kusui // イヘヤビロウドコガネ= iheyabirôdokogane / Maladera (japonica–G) / kusuii / Y. Miyake, 1986 / Det. H. Hirasawa 2012 // Holotype: / Maladera kusuii / Y. MIYAKE, 1986’.

###### Type condition.

The aedeagus of the holotype is pinned separately. The right and left protarsi, the left mesotarsus, and the right and left metatarsi are missing, but some of the missing parts are pinned separately.

###### Current status.

Valid species.

##### Maladera lishana

Taxon classificationAnimaliaColeopteraMelolonthidae

Miyake

4C47BEBD-4B6C-5DCF-9699-F2B9AC54951F

Maladera lishana Miyake, 1989b: 37−38.

###### Note.

The following paratype is deposited in RIEB (ex coll. Y. Miyake):

###### Paratype.

1 ex.: 1 ♀ ‘Lishan / Taiwan / 28. VII. 1973 / Y. Miyake // Paratype: / Maladera / lishana / Y. MIYAKE, 1989’.

###### Current status.

Valid species.

##### Maladera nanshanchiana

Taxon classificationAnimaliaColeopteraMelolonthidae

Nomura

E829D807-21AE-5780-9CA9-5F64D50D126A

Maladera nanshanchiana Nomura, 1974: 111−112.

###### Note.

The following paratypes are deposited in RIEB (ex coll. S. Nomura):

###### Paratypes.

2 exs.: 1 ex. ‘Puli 24 // PARATYPE / Maladera / nanshanchiana / NOMURA (1974)’. 1 ♀ ‘Puli 24 // ♀ // PARATYPE / Maladera / nanshanchiana / NOMURA (1974) // ナンザンケイビロウドコガネ= nanzenkeibirodokogane / Maladera (Cephaloserica) / *nanshanchiana* / Nomura, 1974 / Det. H. Hirasawa 2012’.

###### Current status.

Valid species.

##### Maladera secreta horaiana

Taxon classificationAnimaliaColeopteraMelolonthidae

Nomura

7D4441E9-A5DD-5463-944A-DBA57E2EB4FE

Maladera secreta horaiana Nomura, 1974: 112.

###### Note.

The following paratype is deposited in RIEB (ex coll. S. Nomura):

###### Paratype.

1 ex.: 1 ♂ ‘Lishan / C. Taiwan / 29. vii. 1973 / Y. Miyake // 三宅= miyake // PARATYPE / Maladera / secreta / horaiana / NOMURA (1974)’.

###### Current status.

Valid species.

**Genus *Melanomaladera***

##### Melanomaladera yunnana

Taxon classificationAnimaliaColeopteraMelolonthidae

Miyake & Yamaya

D8508FD0-D29C-59F2-84A4-314045E0FE8D

Melanomaladera yunnana Miyake & Yamaya, 2001: 38−39.

###### Note.

The following paratypes are deposited in RIEB (ex coll. Y. Miyake):

###### Paratypes.

2 exs.: 1 ♂ ‘China, N W Yunnan / Degen City env. / 3300 m alt. / 29. Jun. 1998 / A. Gorodinsky leg. // 13 // Paratype: / Melanoserica / yunnana’. 1 ♀ ‘China, N W Yunnan / Degen City env. / 3300 m alt. / 29. Jun. 1998 / A. Gorodinsky leg. // 13 // Paratype: / Melanoserica / yunnana’.

###### Current status.

*Archeohomaloplia yunnana* (Miyake et Yamaya, 2001), see Ahrens and Bezděk (2016).

###### Remark.

The genus name on the paratype labels is ‘*Melanoserica*’, which is invalid. Probably, it was confused with ‘*Melanomaladera*’ by Y. Miyake.

#### Genus *Microserica*

##### Microserica (Parvulomaladera) annapurnae

Taxon classificationAnimaliaColeopteraMelolonthidae

Ahrens

449910E6-6CA0-5765-AD9B-9C96964C31AA

Microserica ( Parvulomaladera) annapurnae Ahrens, 1995: 46−48.

###### Note.

The following paratype is deposited in RIEB (ex coll. D. Ahrens):

###### Paratype.

1 ex.: ‘NEPAL–HIMALAYA / Annapurna–Mts. / leg. Ahrens 1993 // Pisang bis / Manang, 30. 5. / 3000–3300 m // PARATYPEUS / *Parvulomaladera* / *annapumae sp. n.* / det. D. AHRENS 1994’.

###### Current status.

*Oxyserica pygidialis annapurnae* (Ahrens, 1995), see Ahrens and Bezděk (2016).

##### Microserica nitidipyga

Taxon classificationAnimaliaColeopteraMelolonthidae

Nomura

107FF582-A99F-5A8C-BD61-87C3448AB785

Microserica nitidipyga Nomura, 1974: 99.

###### Note.

The following paratype is deposited in RIEB (ex coll. S. Nomura):

###### Paratype.

1 ex.: ‘Lu-SHAN / FORMOSA / 27. IV. 1973 / K. MATSUDA // 三宅= miyake // Paratype / Microserica / nitidipyga / NOMURA (1974) // Microserica / nitidipyga NOM. / DET. Y. MIYAKE, 1994’.

###### Current status.

Valid species.

#### Genus *Nematophylla*

##### Nematophylla sugiharai

Taxon classificationAnimaliaColeopteraMelolonthidae

Miyake

D799782B-4E7E-58C4-A6B9-710CC7DB1C61

Figure 4E

Nematophylla sugiharai Miyake, 2000: 106−108.

###### Note.

The holotype is deposited in RIEB (ex coll. Y. Miyake):

###### Holotype

**(♂).** ‘Kiau Gap / Mt. Kinabalu / Sabah, Borneo / 1. IV. 2000 / H. SUGIHARAI leg. // Holotype: Nematophylla / sugiharai / Y. MIYAKE 2000’. (Fig. 4E)

###### Current status.

Junior subjective synonym of *Nematophylla carinicollis* Arrow, 1938, see Krajcik (2012).

#### Genus *Nipponoserica*

##### Nipponoserica pubiventris

Taxon classificationAnimaliaColeopteraMelolonthidae

Nomura

B6640F5D-26A5-5BA2-BACB-DF131D0F9C59

Nipponoserica pubiventris Nomura, 1976b: 189−190.

###### Note.

The following paratype is deposited in RIEB (ex coll. S. Nomura):

###### Paratype.

1 ex.: ‘Takashinohama, / Osaka Pref. / 3. VI. 1974 / Coll. Mitsuo Goto // at light // PARATYPE / Nipponoserica / pubiventris / NOMURA (1976)’.

###### Current status.

Valid species.

#### Genus *Paramaladera*

##### Paramaladera makiharai

Taxon classificationAnimaliaColeopteraMelolonthidae

Nomura

0A6553B9-51DD-5B8D-99CC-21368211657C

Paramaladera makiharai Nomura, 1974: 101−102.

###### Note.

The following paratypes are deposited in RIEB (ex coll. S. Nomura):

###### Paratypes.

4 exs.: 4 ♂ ‘Li-shan / Taiwan / 29. VII. 1974 / Y. Miyake // ♂ // Paratype / Paramaladera / makiharai / NOMURA (1974)’.

###### Current status.

Valid species.

#### Genus *Pseudohoplia*

##### Pseudohoplia shibatai

Taxon classificationAnimaliaColeopteraMelolonthidae

Miyake

E6E6C84E-F6DE-556A-AF6B-0096F0C3EFD7

Pseudohoplia shibatai Miyake, 1986b: 201.

###### Note.

The following paratypes are deposited in RIEB (ex coll. Y. Miyake):

###### Paratypes.

2 exs.: 1 ♂ ‘（FORMOSA）/ Mt. Ali / 15 V 1968 / Y. HAYASHI // Paratype // Hoplia / shibataishibatai / Y. Miyake, 1986 / Det. H. Kobayashi, 2015’. 1 ex. ‘（FORMOSA）/ Mt. Ali / 15 V 1968 / Y. HAYASHI // Paratype // Hoplia / shibataishibatai / Y. Miyake, 1986 / Det. H. Kobayashi, 2015’.

###### Current status.

*Hoplia shibatai shibatai* (Miyake, 1986), see Kobayashi (2017).

###### Remark.

In addition to the paratypes mentioned above, the following specimens labeled as paratypes are not designated in the original description: 1 ex. ‘Mt. Arishan / Formosa / 1. V. 1973 / K. MATSUDA // Paratype // Paratype / Pseudohoplia / shibatai / Y. Miyake, 1986 // 贈台湾農試= zou-taiwan-noushi’. 1 ♂ ‘Houanchi- / Sundchuankang / Nantou Hsien / Taiwan / 25. VI. 1976 / H. Makihara leg. // Paratype // Paratype / Pseudohoplia / shibatai / Y. Miyake, 1986’. 1 ex. ‘Mt. Arishan / Formosa / 1. V. 1973 / K. MATSUDA // Paratype / Pseudohoplia / shibatai / Y. Miyake, 1986’. 1 ex. ‘MT. ARI / FORMOSA / 1. V. 1973 / K. MATSUDA // Paratype // Paratype / Pseudohoplia / shibatai / Y. Miyake, 1986’.

##### Pseudohoplia shibatai makiharai

Taxon classificationAnimaliaColeopteraMelolonthidae

Miyake

0A49E652-A2BD-500A-AE0D-D633F9C22127

Figure 4F

Pseudohoplia shibatai makiharai Miyake, 1986b: 202.

###### Note.

The holotype is deposited in RIEB (ex coll. Y. Miyake):

###### Holotype

**(♂).** ‘Mt. Lalashan / Taoyuan Hsien / Taiwan / 21–24. V. 1980 / H. Makihara // HOLOTYPE / Pseudohoplia / shibataimakiharai / Y. MIYAKE, 1886’. (Fig. 4F)

###### Type condition.

The aedeagus of the holotype is pinned separately. The right antenna is missing.

###### Current status.

*Hoplia shibatai makiharai* (Miyake, 1986), see Kobayashi (2017).

##### Pseudohoplia shibatai matsudai

Taxon classificationAnimaliaColeopteraMelolonthidae

Miyake

E4D86FE5-5862-5010-9784-DE4376BD3B2A

Figure 4G

Pseudohoplia shibatai matsudai Miyake, 1986b: 202.

###### Note.

The holotype is deposited in RIEB (ex coll. Y. Miyake):

###### Holotype

**(♂).** ‘Mt. Nōko / C-Formosa / v–20～vi–21’ 66 // HOLOTYPE / Pseudohoplia / shibataimatsudai / Y. MIYAKE, 1986’. (Fig. 4G)

###### Type condition.

The aedeagus of the holotype is pinned separately.

###### Current status.

Junior subjective synonym of *Hoplia shibatai shibatai* (Miyake, 1986), see Kobayashi (2017).

#### Genus *Pseudomaladera*

##### Pseudomaladera nitidifrons

Taxon classificationAnimaliaColeopteraMelolonthidae

Nomura

9DEE74B8-2B9B-59B4-A30C-2F99F861E129

Pseudomaladera nitidifrons Nomura, 1974: 96.

###### Note.

The following paratypes are deposited in RIEB (ex coll. S. Nomura):

###### Paratypes.

2 exs.: 1 ♂ ‘FUNCHIHO / FORMOSA / 1. VIII. 1973 / H. OHASHI // ♂ // 三宅= miyake // Paratype / Pseudomaladera / nitidifrons / NOMURA, 1974’. 1 ♂ ‘MEIFENG / FORMOSA / 1. VIII. 1973 / T. OCHI // ♂ // Paratype / Pseudomaladera / nitidifrons / NOMURA, 1974’.

###### Current status.

Valid species.

#### Genus *Sinoserica*

##### Sinoserica maculipennis

Taxon classificationAnimaliaColeopteraMelolonthidae

Miyake & Yamaya

C96AC3DB-B6FC-5375-ABC4-68BFAC52E78D

Sinoserica maculipennis Miyake & Yamaya, 2001: 40−41.

###### Note.

The following paratypes are deposited in RIEB (ex coll. Y. Miyake):

###### Paratypes.

2 exs.: ‘China, Yunnan / Boshan C. env. / 2100 m alt. / 10. VII. 1998 / A. Gorodinski // 12 // Paratype / Sinoserica / maculipennis’.

###### Current status.

*Trioserica maculipennis* (Miyake et Yamaya, 2001), see Ahrens and Bezděk (2016).

#### Genus *Serica*

##### Serica fusifemorata

Taxon classificationAnimaliaColeopteraMelolonthidae

Nomura

405A8EA4-C8DC-56B6-8F16-5FFB567CD607

Serica fusifemorata Nomura, 1974: 91.

###### Note.

The following paratypes are deposited in RIEB (ex coll. S. Nomura):

###### Paratypes.

11 exs.: 1 ♀ ‘Lishan / C. Taiwan / 29. vii. 1973 / Y. Miyake // ♀ // 三宅= miyake // Paratype / Serica / fusifemorata / NOMURA (1974)’. 2 ♀ ‘Li-shan / C. Taiwan / 26. VII. 1974 / Y. Miyake // ♀ // Paratype / Serica / fusifemorata / S. NOMURA (1974)’. 2 ♂ ‘Li-shan / C. Taiwan / 29. VII. 1974 / Y. Miyake // ♂ // Paratype / Serica / fusifemorata / S. NOMURA (1974)’. 2 ♀ ‘Li-shan / C. Taiwan / 29. VII. 1974 / Y. Miyake // ♀ // Paratype / Serica / fusifemorata / S. NOMURA (1974)’. 1 ♂ ‘Li-shan / C. Taiwan / 1. VIII. 1974 / Y. Miyake // ♂ // Paratype / Serica / fusifemorata / S. NOMURA (1974)’. 2 ♀ ‘Li-shan / C. Taiwan / 1. VIII. 1974 / Y. Miyake // ♀ // Paratype / Serica / fusifemorata / S. NOMURA (1974)’. 1 ♀ ‘27 // ♀ // Paratype / Serica / fusifemorata / S. NOMURA (1974)’.

###### Current status.

Valid species.

##### Serica gansuensis

Taxon classificationAnimaliaColeopteraMelolonthidae

Miyake & Yamaya

26F569ED-6B83-5728-9BFE-D6040B09D546

Serica gansuensis Miyake & Yamaya, 2001: 35−36.

###### Note.

The following paratype is deposited in RIEB (ex coll. Y. Miyake):

###### Paratype.

1 ex.: ‘China, Gangu / Mts. Minshan / nr. Wudn. 2100 / 1. VI. 1997 / A. Gorodinski // 4 // Paratype: / Serica / gansuensis’.

###### Current status.

Valid species.

#### Genus *Sericania*

##### Sericania miyakei

Taxon classificationAnimaliaColeopteraMelolonthidae

Nomura

77BAE2CB-4596-547A-830A-CEF2400A4671

Sericania miyakei Nomura, 1960: 60.

###### Note.

The following paratype is deposited in RIEB (ex coll. S. Nomura):

###### Paratype.

1 ex.: ‘Paratype // TASHIRO / CHIKUGO / 4. V. 1952 / Y. MIYAKE // Sericania / miyakei / NOMURA (1960)’.

###### Current status.

Valid species.

#### Genus *Sophrops*

##### Sophrops takatoshii

Taxon classificationAnimaliaColeopteraMelolonthidae

Itoh

ABB7658D-82E2-5FDA-AFAD-69E263597833

Sophrops takatoshii Itoh, 1990: 9−10.

###### Note.

The following paratypes are deposited in RIEB (ex coll. T. Itoh):

###### Paratypes.

3 exs.: ‘1989–4–17 / 平良植物園= hirara-syokubutsuen /（宮古島）= miyako-jima / leg. T. Ueno // Paratype / Sophrops / takatoshii / T. Itoh’.

###### Current status.

Valid species.

#### Genus *Taiwanoserica*

##### Taiwanoserica elongata

Taxon classificationAnimaliaColeopteraScarabaeidae

Nomura

AAC3C5FA-BDAA-5AC2-AA48-50802597F146

Taiwanoserica elongata Nomura, 1974: 86.

###### Note.

The following paratype is deposited in RIEB (ex coll. S. Nomura):

###### Paratypes.

1 ex.: 1 ♂ ‘MT. ARI / FORMOSA / 30. VII. 1973 / H. OHASHI // ♂ // 三宅= miyake // Paratype / Taiwanoserica / elongata / NOMURA (1974)’.

###### Current status.

Serica (Taiwanoserica) elongata (Nomura, 1974), see Ahrens and Bezděk (2016).

##### Taiwanoserica lishana

Taxon classificationAnimaliaColeopteraScarabaeidae

Nomura

41FA8D7B-9F74-51D4-BA84-DACE70F2825A

Taiwanoserica lishana Nomura, 1974: 87.

###### Note.

The following paratypes are deposited in RIEB (ex coll. S. Nomura):

###### Paratypes.

22 exs.: 2 ♂ ‘Lishan / C. Taiwan / 30. vii. 1970 / Y. Miyake // ♂ // PARATYPE / Taiwanoserica / lishana / S. Nomura (1974)’. 6 ♂ ‘Lishan / C-Taiwan / 29. vii. 1973 / Y. Miyake // ♂ // PARATYPE / Taiwanoserica / lishana / S. Nomura (1974)’. 4 ♀ ‘Lishan / C-Taiwan / 29. vii. 1973 / Y. Miyake // ♀ // PARATYPE / Taiwanoserica / lishana / S. Nomura (1974)’. 4 ♂ ‘Li-shan / Taiwan / 29. VII. 1974 / Y. Miyake // ♂ // PARATYPE / Taiwanoserica / lishana / S. Nomura (1974)’. 1 ♀ ‘Li-shan / Taiwan / 29. VII. 1974 / Y. Miyake // ♀ // PARATYPE / Taiwanoserica / lishana / S. Nomura (1974)’. 4 ♂ ‘Li-shan / Taiwan / 1. VII. 1974 // Y. Miyake // ♂ // PARATYPE / Taiwanoserica / lishana / S. Nomura (1974)’. 1 ♀ ‘Li-shan / Taiwan / 1. VII. 1974 // Y. Miyake // ♀ // PARATYPE / Taiwanoserica / lishana / S. Nomura (1974)’.

###### Current status.

Serica (Taiwanoserica) lishana (Nomura, 1974), see Ahrens and Bezděk (2016).

#### Genus *Trichomaladera*

##### Trichomaladera elongata

Taxon classificationAnimaliaColeopteraMelolonthidae

Nomura

072CD2CC-FE5D-50B0-9BE8-9739964B7400

Trichomaladera elongata Nomura, 1974: 93−94.

###### Note.

The following paratype is deposited in RIEB (ex coll. S. Nomura):

###### Paratype.

1 ex.: 1 ♂ ‘Paratype / Trichomaladera / elongata / NOMURA (1974)’.

###### Current status.

Valid species.

###### Remark.

The collecting data label of the paratype is missing.

#### Genus *Trichoserica*

##### Trichoserica elongata nitididorsis

Taxon classificationAnimaliaColeopteraScarabaeidae

Nomura

0D69CA90-4BAE-5465-B80F-DBB1798A5E28

Trichoserica elongata nitididorsis Nomura, 1971: 70−71.

###### Note.

The following paratype is deposited in RIEB (ex coll. S. Nomura):

###### Paratype.

1 ex.: ‘Mt. Hiko / [KYUSHU] / 27. 7. 1960 / TAKEISHI // Paratype / Trichoserica / elongata / nitididorsis / Nomura’.

###### Current status.

*Serica nitididorsis* (Nomura, 1971), see Ahrens and Bezděk (2016).

##### Trichoserica incurvata

Taxon classificationAnimaliaColeopteraScarabaeidae

Nomura

937C9036-527A-55E4-8EE3-B8B533B6C78C

Trichoserica incurvata Nomura, 1971: 67−68.

###### Note.

The following paratypes are deposited in RIEB (ex coll. S. Nomura):

###### Paratypes.

8 exs.: 1 ex. ‘Aomori-pref / 17–vii–1956 / S. Kimotog // Paratype / Trichoserica / incurvata / Nomura (1971) // Aomori / 17. VII. 1956 / S. Kimoto’. 1 ♂ ‘17. VII. 1956 / S. Kimoto // Paratype / Trichoserica / incurvata / Nomura (1971)’. 2 exs. ‘Aomori-pref / 19–vii–1956 / S. Kimotog // Paratype / Trichoserica / incurvata / Nomura (1971) // Kimoto S. / 19. VII. 1956’. 1 ex. ‘Aomori-pref / 19–vii–1956 / S. Kimotog // Paratype / Trichoserica / incurvata / Nomura (1971) // 19. VII. 1956 / S. Kimoto’. 2 exs. ‘Sensuidani 1200 m / Okutama Tokyo / 21–VII–1968 / Coll. H. Yuasa // Paratype / Trichoserica / incurvata / Nomura (1971)’. 1 ex. ‘Okutama / 21. VII. 1968 / YUASA // Paratype / Trichoserica / incurvata / Nomura (1971)’.

###### Current status.

*Serica incurvata* (Nomura, 1971), see Ahrens and Bezděk (2016).

##### Trichoserica nitidiceps

Taxon classificationAnimaliaColeopteraScarabaeidae

Nomura

CCC606C9-2E36-55D4-AEA2-F3DD74E683E9

Trichoserica nitidiceps Nomura, 1971: 69−70.

###### Note.

The following paratype is deposited in RIEB (ex coll. S. Nomura):

###### Paratype.

1 ex.: 1 ♂ ‘(Kyushu) / Mt. Sobo / 23. V. 1952 / Y. Miyake //Paratype / Trichoserica / nitidiceps / NOMURA’.

###### Current status.

Junior subjective synonym of *Serica inexspectata* (Kontkanen, 1956), see Ahrens and Bezděk (2016).

###### Remark.

In addition to the paratype mentioned above, the following specimen labeled as paratype is not designated in the original description: 1 ex. ‘Mt. Kuju / Bungo / 28. vii. 1961 / Y. Miyake // Paratype / Trichoserica / nitidiceps / NOMURA (1971)’.

##### Trichoserica opacifrons

Taxon classificationAnimaliaColeopteraScarabaeidae

Nomura

4489A415-A7B2-5974-9793-05208846DC21

Trichoserica opacifrons Nomura, 1971: 68−69.

###### Note.

The following paratypes are deposited in RIEB (ex coll. S. Nomura):

###### Paratypes.

3 exs.: 1 ♂ ‘Mt. Hiko / Buzen / 1. VIII. 1953 / Y. Miyake // Paratype / Trichoserica / opacifrons / NOMURA’. 1 ex. ‘mt. Kujū / Hokkeein / 27. VII. 1961 / Y. Miyake Coll // Paratype / Trichoserica / opacifrons / NOMURA’. 1 ♂ ‘ [Kyushu] / Yakushima / 21. Vi. 1954 / Col. K. Matsuda // Paratype / Trichoserica / opacifrons / NOMURA // Ophthalmoserica/ matsudai / sp.n. / DET. Y. MIYAKE’.

###### Current status.

Senior objective synonym of *Serica planifrons* Nomura, 1972, see Ahrens and Bezděk (2016). Nomura (1972) proposed *Serica planifrons* as a replacement name for *Serica opacifrons* (Nomura, 1971), a junior secondary homonym of *Serica opacifrons* Fairmaire, 1891.

#### Subfamily Cetoniinae

**Genus *Anomalocera***

##### Anomalocera paotao

Taxon classificationAnimaliaColeopteraPontellidae

Masumoto & Sakai

357351E6-02D3-5EC6-80C1-0E7BBE2F3540

Anomalocera paotao Masumoto & Sakai, 1987a: 51−54.

###### Note.

The following paratypes are deposited in RIEB (ex coll. Masumoto):

###### Paratypes.

2 exs.: 1 ex. ‘Lishan / Taiwan / 28. VII. 1974 / Y. Miyake // Paratype / Anomalocera / paotao MAS. et SAK.’. 1 ex. ‘Lishan / C, -Taiwan / 29 vii. 1973 / Y. Miyake // Paratype / Anomalocera / paotao MAS. et Sak.’.

###### Current status.

*Diphyllomorpha paotao* (Masumoto et Sakai, 1987), see Bezděk (2016).

#### Genus *Rhomborhina*

##### Rhomborhina kurosawai

Taxon classificationAnimaliaColeopteraCetoniidae

Masumoto & Sakai

1AEF134E-A348-500D-8AA3-6B909D4DBB88

Rhomborhina kurosawai Masumoto & Sakai, 1987b: 104−105, 107.

###### Note.

The following paratypes are deposited in RIEB (ex coll. Masumoto):

###### Paratypes.

3 exs.: 2 ♂ ‘FORMOSA / tattaka / 9, VIII 1971 / H. OHARA // Paratype / Rhomborrhina / kurosawai / MASUMOTO et K. SAKAI’. [[1 ♂ ‘Tsuifeng / C. Tsiwan / 26. vii. 1970 (the date given in the original description is 27.vii.1970) // Y. Miyake // Paratype / Rhomborrhina / kurosawai / MASUMOTO et K. SAKAI’]].

###### Current status.

Valid species.

#### Genus *Thaumastopeus*

##### Thaumastopeus chicheryi tenuigenius

Taxon classificationAnimaliaColeopteraScarabaeidae

Miyake & Yamaya

2DC8B93A-3ED6-5DC0-ACF5-A8D49E5303A0

Thaumastopeus chicheryi tenuigenius Miyake & Yamaya, 1995: 38.

###### Note.

The following paratype is deposited in RIEB (ex coll. Y. Miyake):

###### Paratype.

1 ex.: 1 ♂ ‘N. Keningau / N. Borneo / 30. V. 1994 // Paratype: Thaumastopeus / chicheryitenuigenius / Y. MIYAKE et YAMAYA, 1995’.

###### Current status.

Valid species.

#### Genus *Paratrichius*

##### Paratrichius hatai

Taxon classificationAnimaliaColeopteraCetoniidae

Miyake

3BDD0267-EC48-5C35-B9A4-B703F440D8A9

Paratrichius hatai Miyake, 1989: 41−42.

###### Note.

The following paratypes are deposited in RIEB (ex coll. Y. Miyake):

###### Paratypes.

4 exs.: [[‘Tanah Rata / MALAYA / 7. IV. 1972 (the date given in the original description is 7.IV.1978) / M. Hata // Paratype: / Paratrichius / hatai / Y. MIYAKE, 1989’]].

###### Current status.

Valid species.

##### Paratrichius kyushuensis

Taxon classificationAnimaliaColeopteraCetoniidae

Miyake

E5E0CDE7-4F2C-5542-BDAF-A447CA3897D0

Figure 4H

Paratrichius kyushuensis Miyake, 1990: 30−32.

###### Note.

The holotype and following paratypes are deposited in RIEB (ex coll. Y. Miyake):

###### Holotype

**(♂).** ‘MT. SOBO / BUNGO / 28. vii. 1954 / Y. MIYAKE // Holotype: / Paratrichius / kyushuensis / Y. MIYAKE, 1990’. (Fig. 4H)

###### Paratypes.

5 exs.: 1 ex. ‘(Kyushu) / Mt. Sobo / 24. VII. 1950 / Y. Miyake // Paratype: Paratrichius / kyushuensis / Y. MIYAKE, 1990’. 1 ♂ ‘(Kyushu) / Mt. Sobo / 24. VII. 1952 / Y. Miyake // Paratype: Paratrichius / kyushuensis / Y. MIYAKE, 1990’. 1 ex. ‘(Kyushu) / Mt. Ichi Fu Sa / 28 VII 1952 / Col. S. Kimoto // Paratype: Paratrichius / kyushuensis / Y. MIYAKE, 1990’. 1 ex. 1 ♂ ‘(Kyushu) / Mt. Sobo / 24. VII. 1953 / K. MATSUDA // Paratype: Paratrichius / kyushuensis / Y. MIYAKE, 1990’.

###### Type condition.

The left metatarsus of the holotype is missing.

###### Current status.

Valid species.

###### Remark.

The date on the label of the holotype is 28.VII.1954 while that in the original description is 28.VII.1952.

In addition to the paratypes mentioned above, the following specimen labeled as paratype is not designated in the original description.: 1 ex. ‘⑦ // Paratype: Paratrichius / kyushuensis / Y. MIYAKE, 1990’.

#### Genus *Tibiotrichius*

##### Tibiotrichius vietnamensis

Taxon classificationAnimaliaColeopteraCetoniidae

Miyake

EBC2D9E6-06B4-5EDD-BD77-EFDD01573701

Figure 5A

Tibiotrichius vietnamensis Miyake, 1996: 44.

###### Note.

The holotype is deposited in RIEB (ex coll. Y. Miyake):

###### Holotype

**Figure 5.**
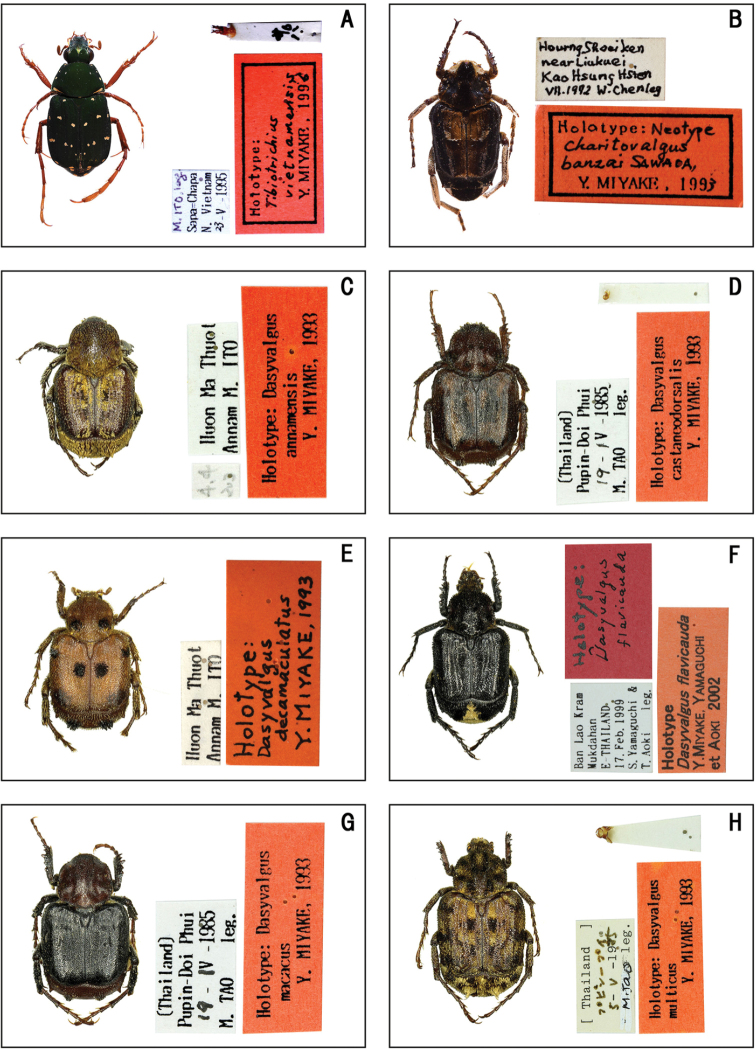
Habitus of holotype specimens. **A***Tibiotrichius vietnamensis* Miyake **B***Charitovalgus banzai* Sawada **C***Dasyvalgus annamensis* Miyake **D***Dasyvalgus castaneodorsalis* Miyake **E***Dasyvalgus decamaculatus* Miyake **F***Dasyvalgus flavicauda* Miyake **G***Dasyvalgus macacus* Miyake **H***Dasyvalgus multicus* Miyake.

**(♂).** ‘M. ITO leg / Sapa=Chapa / N. Vietnam / 23–V–1995 // サパ= sapa [aedeagus mount] // Holotype: Tibiotrichius / vietnamensis Y. MIYAKE, 1996’. (Fig. 5A)

###### Type condition.

The aedeagus of the holotype is pinned separately.

###### Current status.

Valid species.

#### Subfamily Valginae

**Genus *Charitovalgus***

##### Charitovalgus banzai

Taxon classificationAnimaliaColeopteraCetoniidae

Sawada

C1A03233-EFFC-5E3F-96CD-37A074F67167

Figure 5B

Charitovalgus banzai Sawada, 1941: 8−9.

###### Note.

The neotype is deposited in RIEB (ex coll. Y. Miyake):

###### Neotype

**(♂).** ‘Hourng Shoei ken / near Liukuei, / Kao Hsung Hsien / VII. 1972 W. Chen leg // Holotype: Neotype / Charitovalgus / banzai SAWADA / Y. MIYAKE, 1993’. (Fig. 5B)

###### Type condition.

The right and left metatarsi of the neotype are missing.

###### Current status.

Valid species.

###### Remark.

The neotype was designated by Miyake (1993).

#### Genus *Dasyvalgus*

##### Dasyvalgus annamensis

Taxon classificationAnimaliaColeopteraCetoniidae

Miyake

F621BF50-71E8-5912-AB11-752C31A172A3

Figure 5C

Dasyvalgus annamensis Miyake, 1993a: 21−22.

###### Note.

The holotype and following paratype are deposited in RIEB (ex coll. Y. Miyake):

###### Holotype

**(♂).** ‘4. 4 / 203 // Iluon Ma Thuot / Annam M. ITO // Holotype: Dasyvalgus / Annamensis / Y. MIYAKE, 1993’. (Fig. 5C)

###### Paratype.

1 ex.: 1 ♂ ‘Iluon Ma Thuot / Annam M. ITO // Paratype: Dasyvalgus / annamensis / Y. MIYAKE, 1993’.

###### Current status.

Valid species.

###### Remark.

The locality on the collecting data label of the holotype is ‘Iluon Ma Thuot’, but the locality given in the original description is ‘Ban Me Thuot’. However, the habitus of the holotype and the figure in the original description match. Probably, Miyake made a typographical error when writing the description.

##### Dasyvalgus castaneodorsalis

Taxon classificationAnimaliaColeopteraCetoniidae

Miyake

A920D72A-FEBB-5DF3-B0B4-8F68D6F9C77F

Figure 5D

Dasyvalgus castaneodorsalis Miyake, 1993a: 24−25.

###### Note.

The holotype and following paratypes are deposited in RIEB (ex coll. Y. Miyake):

###### Holotype

**(♂).** ‘〔Thailand〕/ Pupin–Doi Phui / 19–IV–1985 / M. TAO leg. // Holotype: Dasyvalgus / casraneodorsalis / Y. MIYAKE, 1993’. (Fig. 5D)

###### Paratypes.

6 exs.: 2 ♂ ‘〔Thailand〕/ Pupin-Doi-Phui / 19–IV–1985 / M. TAO leg. // Paratype: Dasyvalgus / castaneodorsalis / Y. MIYAKE, 1993’. 1 ♂ ‘〔Thailand〕/ Pupin-Doi-Phui / 21–IV–1985 / M. TAO leg. // Paratype: Dasyvalgus / castaneodorsalis / Y. MIYAKE, 1993’. 2 ♂ ‘〔Thailand〕/ Pupin / 21–IV–1985 / M. TAO // Paratype: Dasyvalgus / castaneodorsalis / Y. MIYAKE, 1993’. 1 ♂ ‘〔Thailand〕/ Doi Suthep / 10–V–1985 / M. TAO leg. // Paratype: Dasyvalgus / castaneodorsalis / Y. MIYAKE, 1993’.

###### Type condition.

The aedeagus of the holotype is pinned separately.

###### Current status.

Valid species.

##### Dasyvalgus decamaculatus

Taxon classificationAnimaliaColeopteraCetoniidae

Miyake

009FA200-B1A7-5663-B4A4-13E3612B57D2

Figure 5E

Dasyvalgus decamaculatus Miyake, 1993a: 22−23.

###### Note.

The holotype and following paratypes are deposited in RIEB (ex coll. Y. Miyake):

###### Holotype

**(♂).** ‘Iluon Ma Thuot / Annam M. ITO // Holotype: / Dasyvalgus / decamaculatus / Y. MIYAKE, 1993’. (Fig. 5E)

###### Paratypes.

2 exs.: 1 ex. ‘Near Chang Mai / N. Thailand / 7–V–1994 / K. KUME leg. // Paraype: Dasyvalgus / decamaculatus / Y. MIYAKE, 1993’. 1 ex. ‘Paratype: Dasyvalgus / decamaculatus / Y. MIYAKE, 1993’.

###### Type condition.

The right and left metatarsus are missing.

###### Current status.

Valid species.

###### Remark.

The locality on the collecting data label of the holotype is ‘Iluon Ma Thuot’, but the locality given in the original description is ‘Ban Me Thuot’. However, the habitus of the holotype and the figure in the original description match. Probably, Miyake caused a typographical error when writing the description.

##### Dasyvalgus flavicauda

Taxon classificationAnimaliaColeopteraCetoniidae

Miyake, Yamaguchi et Aoki

71C09282-C078-521A-B055-8FCAA7DAD4C3

Figure 5F

Dasyvalgus flavicauda Miyake, Yamaguchi, Aoki et Akiyama, 2002: 65−66.

###### Note.

The holotype and following paratype are deposited in RIEB (ex coll. Y. Miyake):

###### Holotype

**(♂).** ‘Ban Lao Kram / Mukdahan / E-THAILAND / 17. Feb. 1999 / S. Yamaguchi and / T. Aoki leg. // Holotype: / Dasyvalgus / flavicauda // Holotype / Dasyvalgus flavicauda / Y. MIYAKE, YAMAGUCHI / et AOKI 2002’. (Fig. 5F)

###### Paratype.

1 ex.: 1 ♂ ‘Phu Huai Sing / Mukdahan / E–THAILAND / 19. Feb. 1999 / S. Yamaguchi and / T. Aoki leg. // Paratype / Dasyvalgus flavicauda / Y. MIYAKE, YAMAGUCHI / et AOKI 2002’.

###### Current status.

Valid species.

##### Dasyvalgus macacus

Taxon classificationAnimaliaColeopteraCetoniidae

Miyake

3E894F15-AE19-5519-A811-43E6A8ABA179

Figure 5G

Dasyvalgus macacus Miyake, 1993a: 24.

###### Note.

The holotype and following paratypes are deposited in RIEB (ex coll. Y. Miyake):

###### Holotype

**(♂).** ‘〔Thailand〕Pupin–Doi Phui / 19–IV–1985 / M. TAO leg. // Holotype: Dasyvalgus / macacus / Y. MIYSKE, 1993’. (Fig. 5G)

###### Paratypes.

4 exs.: 1 ♂ ‘〔Thailand〕/ Pupin-Doi Phui / 1–V–1985 / M. TAO leg. // Paratype: Dasyvalgus / macacus / Y. MIYAKE, 1993’. 1 ♂ ‘〔Thailand〕/ Pupin-Doi Phui / 5–V–1985 / M. TAO leg. // Paratype: Dasyvalgus / macacus / Y. MIYAKE, 1993’. 1 ♂ ‘〔Thailand〕/ Pupin-Doi Phui / 12–V–1985 / M. TAO leg. // Paratype: Dasyvalgus / macacus / Y. MIYAKE, 1993’. 1 ♂ ‘〔Thailand〕/ Pupin-Doi Phui / 21–V–1985 / M. TAO leg. // Paratype: Dasyvalgus / macacus / Y. MIYAKE, 1993’.

###### Current status.

Valid species.

###### Remark.

The habitus photograph in the original description does not refer to the holotype.

##### Dasyvalgus makiharai

Taxon classificationAnimaliaColeopteraCetoniidae

Miyake

B2A5823C-EA37-57E7-97DA-D57420920DCA

Dasyvalgus makiharai Miyake, 1985: 11−12.

###### Note.

The following paratypes are deposited in RIEB (ex coll. Y. Miyake):

###### Paratype.

1 ex.: ‘MEIFENG / FORMOSA / 3–VII. 74 / T, OCHI // Paratype: / Dasyvalgus / makiharai Y. MIYAKE / (1985)’.

###### Current status.

Valid species.

###### Remark.

In addition to the paratype mentioned above, the following specimens labeled as paratype are not designated in the original description: 1 ex. ‘松崗= songgang / 27～29. VII 1968 / K. YAMAMOTO // Paratype: Dasyvalgus / makiharai / Y. MIYAKE, 1985’. 1 ♂ ‘SUNG KANG / FORMOSA / 30–VI, 74 / T. OCHI // Paratype: / Dasyvalgus / makiharai Y. MIYAKE / (1985)’.

##### Dasyvalgus minahasanus

Taxon classificationAnimaliaColeopteraCetoniidae

Miyake

A4E3A16F-21E7-594C-B01D-F5F9183FB20D

Dasyvalgus minahasanus Miyake, 1989b: 42−43.

###### Note.

The following paratypes are deposited in RIEB (ex coll. Y. Miyake):

###### Paratypes.

78 exs.: 75 exs. ‘Tondano / N. Sulawesi / 10–IV–1989 / Y. Miyake leg. // Paratype: / Dasyvalgus / minahasanus / Y. MIYAKE, 1989’. 3 exs. ‘Tondano / N. Sulawesi / 9–IV–1989 / Y. Miyake leg. // Paratype: / Dasyvalgus / minahasanus / Y. MIYAKE, 1989’.

###### Current status.

Valid species.

###### Remark.

In addition to the paratypes mentioned above, the following specimen labeled as paratypes is not designated in the original description: 1 ex. ‘Tondano / N. Sulawesi / 11–IV–1989 / Y. Miyake leg. // Paratype: / Dasyvalgus / minahasanus / Y. MIYAKE, 1989’.

##### Dasyvalgus multicus

Taxon classificationAnimaliaColeopteraCetoniidae

Miyake

415E32BB-7257-567E-BC1A-24AB1AF83AD6

Figure 5H

Dasyvalgus multicus Miyake, 1993a: 25−26.

###### Note.

The holotype is deposited in RIEB (ex coll. Y. Miyake):

###### Holotype

**(♂).** ‘ [Thailand] / プピン-プイ= pupin-pui / 5–V–1985 / M. Tao leg. // Holotype: Dasyvalgus / multicus / Y. MIYAKE, 1993’. (Fig. 5H)

###### Type condition.

The aedeagus of the holotype is pinned separately. The right and left protarsus are missing.

###### Current status.

Valid species.

##### Dasyvalgus nudis

Taxon classificationAnimaliaColeopteraCetoniidae

Miyake

E38711E1-4687-505C-8356-B3418077BE07

Figure 6A

Dasyvalgus nudis Miyake, 1994: 153.

###### Note.

The holotype and following paratypes are deposited in RIEB (ex coll. Y. Miyake):

###### Holotype

**Figure 6.**
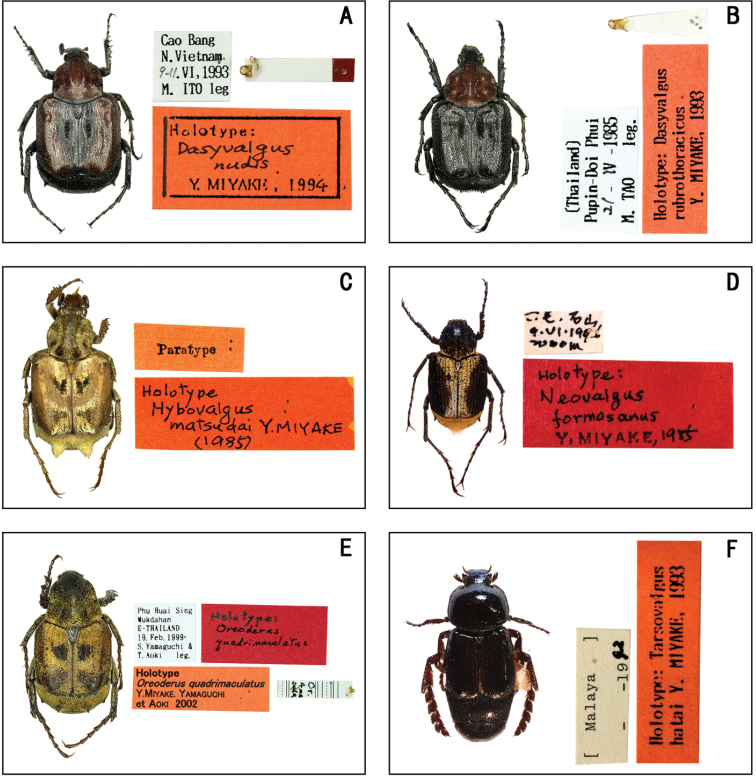
Habitus of holotype specimens. **A***Dasyvalgus nudis* Miyake **B***Dasyvalgus rubrothoracicus* Miyake **C***Hybovalgus matsudai* Miyake **D***Neovalgus formosanus* Miyake **E***Oreoderus quadrimaculatus* Miyake **F***Tarsovalgus hatai* Miyake.

**(♂).** ‘Cao Bang / N. Vietnam / 9–11. VI, 1993 / M. ITO leg // Holotype: Dasyvalgus / nudis / Y. MIYAKE, 1994’. (Fig. 6A)

###### Paratypes.

9 exs.: 3 ♂ ‘Cao Bang / N. Vietnam / 8–10 VI, 1993 / M. ITO leg. // Paratype: / Dasyvalgus / nudis / Y. MIYAKE, 1994’. 3 ♂ ‘Cao Bang / N. Vietnam / 10. VI, 1993 / M. ITO leg. // Paratype: / Dasyvalgus / nudis / Y. MIYAKE, 1994’. [[2 ♂ ‘Cao Bang / N. Vietnam / 9–11. VI, 1993 (real date is 8–10. VI. 1993) / M. ITO leg. // Paratype: / Dasyvalgus / nudis / Y. MIYAKE, 1994’]]. [[1 ex. ‘Cao Bang / N. Vietnam / 11. VI, 1993 (real date is 8–10. VI. 1993) / M. ITO leg. // Paratype: / Dasyvalgus / nudis / Y. MIYAKE, 1994’]].

###### Type condition.

The aedeagus of the holotype is pinned separately. The right antenna is missing.

###### Current status.

Valid species.

###### Remark.

The date on the collecting data label of the holotype is 9–11,VI,1993, but the collecting date quoted in the original description is 8–10,VI,1993. However, the habitus of the holotype and the figure in the original description match. Probably, Miyake caused a typographical error when writing the description.

##### Dasyvalgus rubrothoracicus

Taxon classificationAnimaliaColeopteraCetoniidae

Miyake

A362D698-39B2-5BBA-9145-1F0E32E71F1E

Figure 6B

Dasyvalgus rubrothoracicus Miyake, 1993a: 23−24.

###### Note.

The holotype and following paratypes are deposited in RIEB (ex coll. Y. Miyake):

###### Holotype

**(♂).** ‘〔Thailand〕/ Pupin–Doi Phui / 21–IV–1985 / M. TAO leg. // Holotype: Dasyvalgus / rubrothoracicus / Y. MIYAKE, 1993’. (Fig. 6B)

###### Paratypes.

11 exs.: 1 ex. ‘Doi Sutehp / Thailand / 28–IV–1985 / M Tao leg. // TDvA // Paratype: Dasyvalgus / rubrothoracicus / Y. MIYAKE, 1993’. 1 ♂ ‘〔Thailand〕/ Pupin-Doi-Phui / 1–V–1985 / M. TAO leg. // Paratype: Dasyvalgus / rubrothoracicus / Y. MIYAKE, 1993’. 1 ♂ ‘〔Thailand〕/ Pupin-Doi-Phui / 1–V–1985 / M. TAO leg. // TDvA // Paratype: Dasyvalgus / rubrothoracicus / Y. MIYAKE, 1993’. 1 ♂ ‘〔Thailand〕/ Pupin-Doi-Phui / 5–V–1985 / M. TAO leg. // TDvA // TDvA // Paratype: Dasyvalgus / rubrothoracicus / Y. MIYAKE, 1993’. 1 ♂ ‘〔Thailand〕/ Pupin-Doi-Phui / 11–V–1985 / M. TAO leg. // Paratype: Dasyvalgus / rubrothoracicus / Y. MIYAKE, 1993’. [[1 ♂ ‘〔Thailand〕/ Pupin-Doi-Phui / 17–V–1985 (the date given in the original description is 17–IV–1985) / M. TAO leg. // Paratype: Dasyvalgus / rubrothoracicus / Y. MIYAKE, 1993’]]. [[1 ♂ ‘〔Thailand〕/ Pupin-Doi-Phui / 21–V–1985 (the date given in the original description is 21–IV–1985) / M. TAO leg. // TDvA // Paratype: Dasyvalgus / rubrothoracicus / Y. MIYAKE, 1993’]]. [[1 ♂ ‘〔Thailand〕/ Pupin-Doi-Phui / 21–V–1985 (the date given in the original description is 21–IV–1985) / M. TAO leg. // TDvA // Paratype: Dasyvalgus / rubrothoracicus / Y. MIYAKE, 1993’]]. [[1 ♂‘〔Thailand〕/ Pupin-Doi-Phui / 23–V–1985 (the date given in the original description is 23–IV–1985) / M. TAO leg. // Paratype: Dasyvalgus / rubrothoracicus / Y. MIYAKE, 1993’]]. [[2 ♂ ‘〔Thailand〕/ Pupin-Doi-Phui / 28–V–1985 (the date given in the original description is 28–IV–1985) / M. TAO leg. // TDvA // Paratype: Dasyvalgus / rubrothoracicus / Y. MIYAKE, 1993’]].

###### Type condition.

The aedeagus of the holotype is pinned separately.

###### Current status.

Valid species.

###### Remark.

The date on the collecting data label of the holotype is 21–IV–1985, but the collecting date quoted in the original description is 5–V–1985. However, the habitus of the holotype and the figure in the original description match. Probably, Miyake caused a typographical error when writing the description.

##### Dasyvalgus similis

Taxon classificationAnimaliaColeopteraCetoniidae

Miyake

5167838A-9A15-596E-A172-79D561BA425F

Dasyvalgus similis Miyake, 1985: 8−9.

###### Note.

The following paratypes are deposited in RIEB (ex coll. Y. Miyake):

###### Paratypes.

3 exs.: 1 ♂ ‘蓬来= hôrai / 20, 21 VIII 1968 / H. MAKIHARA // Paratype’. 1 ♂ ‘FUNCHIIFO / FORMOSA / 1. VI. 1970 / Y. KIYOYAMA // Paratype // Paratype / Dasyvalgus / similis, Y. MIYAKE / 1985’. 1 ♂ ‘FUNCHIIFO / FORMOSA / 1. VI. 1970 / Y. KIYOYAMA // Paratype’.

###### Current status.

Senior objective synonym of *Dasyvalgus taiwanus* Miyake, 1991. Miyake in Miyake et al. (1991) proposed *Dasyvalgus taiwanus* as a replacement name for *Dasyvalgus similis* Miyake, 1985, a junior primary homonym of *Dasyvalgus similis* Moser, 1908.

###### Remark.

In addition to the paratypes mentioned above, the following specimens labeled as paratypes are not designated in the original description: 1 ex. ‘LIUKUEI / FORMOSA / 3. X. 1970 / Y. KIYOYAMA // 16 // Paratype // Paratype / Dasyvalgus / similis Y. MIYAKE, 1985’. 1 ex. ‘NANSHANCHI / FORMOSA / 27. VI. 1971 / Y. MAEDA // Paratype // Paratype / Dasyvalgus / similis Y. MIYAKE, 1985’. 1 ex. ‘紅水坑= hongshuikeng / Liukuei / 11. V. 1978 / Chen. W. // Paratype’. 1 ♂ ‘紅水坑= hongshuikeng / Liukuei / 11. V. 1978 / Chen. W. // Paratype’. 1 ex. ‘紅水坑= hongshuikeng / Liukuei / 11. V. 1978 / W. Chen // Paratype // Paratype / Dasyvalgus / similis Y. MIYAKE, 1985’. 1 ex. ‘Paratype’.

##### Dasyvalgus wadai

Taxon classificationAnimaliaColeopteraCetoniidae

Miyake

D0B3AFA2-D024-57A6-A144-F4C74A23B05D

Dasyvalgus wadai Miyake, 1985: 9−10.

###### Note.

The following paratype is deposited in RIEB (ex coll. Y. Miyake):

###### Paratype.

1 ex.: 1 ♂ ‘Paratype / Dasyvalgus / wadai Y. MIYAKE, 1985’.

###### Current status.

Valid species.

###### Remark.

This paratype has no collecting data label.

#### Genus *Hybovalgus*

##### Hybovalgus matsudai

Taxon classificationAnimaliaColeopteraCetoniidae

Miyake

4EA313E3-E144-5B78-9B36-AA5A92447B6E

Figure 6C

Hybovalgus matsudai Miyake, 1985: 6.

###### Note.

The holotype is deposited in RIEB (ex coll. Y. Miyake):

###### Holotype

**(♂).** ‘Paratype // Holotype / Hybovalgus / matsudai Y. MIYAKE / (1985)’.

###### Type condition.

The right protarsus is missing.

###### Current status.

Valid species.

###### Remark.

There is no data label attached to the holotype, but the habitus of the holotype matches the figure in the original description. If confidence is placed in the pinned holotype label and the habitus, the above holotype specimen is the true holotype.

#### Genus *Neovalgus*

##### Neovalgus formosanus

Taxon classificationAnimaliaColeopteraCetoniidae

Miyake

1B144BD3-2306-5C0A-837F-1049679B8679

Figure 6D

Neovalgus formosanus Miyake, 1985: 3.

###### Note.

The holotype and following paratypes are deposited in RIEB (ex coll. Y. Miyake):

###### Holotype

**(♂).** ‘六亀= liugui．石山= shi shan / 9. VI. 1976 / 2000m // Holotype: / Neovalgus / formosanus / Y, MIYAKE, 1985’. (Fig. 6D)

###### Paratypes.

2 exs.: 1 ex. ‘ [Taiwan] / Baibara, mt. Puli / –V–1965 // Paratype: Neovalgus / formosanus Y. MIYAKE, 1985’. 1 ex. ‘ [Taiwan] / Liukuei / 16–V–1978 // PARATYPE / Neovalgus / formosanus / Y. MIYAKE, 1985’.

###### Current status.

Valid species.

###### Remark.

The date on the collecting label of the holotype is 9.VI.1976, but the collecting date quoted in the original description is 16.V.1978. However, the habitus of the holotype matches the figure in the original description. Probably, Miyake caused a typographical error when writing the description.

In addition to the paratypes mentioned above, the following specimens labeled as paratypes are not designated in the original description: 3 exs. ‘ [Taiwan] / Baibara, mt. Puli / –V–1965 // PARATYPE / Neovalgus / formosanus / Y. MIYAKE, 1985’. 1 ex. ‘Formosa / Wushe / V. 1965 / T. Shiroz / Paratype: Neovalgus / formosanus / Y. MIYAKE, 1985’. 1 ex. ‘六 亀= liugui, 石山= shi shan / 2000 m / 9–VI–1976 / Paratype / Neovalgus / formosanus / Y. MIYAKE, 1985’. 1 ex. ‘Paratype: / Neovalgus / formosanus / Y. MIYAKE, 1985’.

#### Genus *Oreoderus*

##### Oreoderus quadrimaculatus

Taxon classificationAnimaliaColeopteraCetoniidae

Miyake, Yamaguchi et Aoki

C82B01EE-7E4C-5CE6-80B0-417B8A698CE0

Figure 6E

Oreoderus quadrimaculatus Miyake, Yamaguchi, Aoki et Akiyama, 2002: 64−65.

###### Note.

The holotype is deposited in RIEB (ex coll. Y. Miyake):

###### Holotype

**(♂).** ‘Phu Huai Sing / Mukdahan / E-THAILAND / 19. Feb. 1999 / S. Yamahuchi and T. Aoki leg. // Holotype: / Oreoderus / quadrumaculatus // Or. 4ml / Coll. // Holotype / Oreoderus quadrimaculatus / Y. MIYAKE, YAMAGUCHI / et AOKI 2002’. (Fig. 6E)

###### Type condition.

The aedeagus of the holotype is pinned separately. The right protarsus is missing.

###### Current status.

Valid species.

#### Genus *Tarsovalgus*

##### Tarsovalgus hatai

Taxon classificationAnimaliaColeopteraCetoniidae

Miyake

67027003-E0F6-5F5B-875B-72327D176BFF

Figure 6F

Tarsovalgus hatai Miyake, 1993b: 32−33.

###### Note.

The holotype and following paratype are deposited in RIEB (ex coll. Y. Miyake):

###### Holotype

**(♂).** ‘ [Malaya] / – –1992 // Holotype: Tarsovalgus / hatai Y. MIYAKE, 1993’. (Fig. 6F)

###### Paratype.

1 ex.: ‘ [Malaya] / – –1992 // Paratype: / Tarsovalgus / hatai / Y. MIYAKE, 1993’.

###### Current status.

Valid species.

### Family Trogidae

#### Genus *Trox*

##### Trox kyotensis

Taxon classificationAnimaliaColeopteraTrogidae

Ochi & Kawahara

5D0F00DB-1B88-5D3F-812D-B59FE8084411

Trox kyotensis Ochi & Kawahara, 2000: 53−56.

###### Note.

The following paratype is deposited in RIEB (ex coll. T. Ochi):

###### Paratype.

1 ex.: ‘YAMAZAKI- / CHO, KYOTO. / 5–IV, 1999 / M. KAWAHARA // PARATYPE / Trox / kyotensis / OCHI and KAWAHARA / 2000’.

###### Current status.

Trox (Niditrox) kyotensis Ochi et Kawahara, 2000, see Kalz (2019).

##### Trox sabulosus fujiokai

Taxon classificationAnimaliaColeopteraTrogidae

Ochi

F2C2474D-E5CB-5695-B111-A24B8FE255AB

Trox sabulosus fujiokai Ochi, 2000: 43−44.

###### Note.

The following paratype is deposited in RIEB (ex coll. T. Ochi):

###### Paratype.

1 ex.: ‘Yukyuzan-Park, Nagaoka / Niigata Pref., Japan / 28. V. 1997 / Y and M. Kawahara leg // PARATYPE / Trox / Sabulosus / Subsp. fujiokai / OCHI, 2000’.

###### Current status.

Valid species.

##### Trox setifer horiguchii

Taxon classificationAnimaliaColeopteraTrogidae

Ochi & Kawahara

CB4CF742-61BA-501D-90E6-D88EB3DCFBCF

Trox setifer horiguchii Ochi & Kawahara, 2002: 54−55.

###### Note.

The following paratype is deposited in RIEB (ex coll. T. Ochi):

###### Paratype.

1 ex.: 1♂ ‘MEBORO-DAM / KAMIAGATA. T. / IS. TSUSHIMA / NAGASAKI. J. P / 25–V. 2002 / Y and M. KAWAHARA // 対県= tsu-ken上県町= kamiagata-chô / 目保呂ダム= meborodamu / 25. V. 2002 / Y and M. Kawahara // ♂ // PARATYPE / Trox / setifer / subsp. horiguchii / OCHI and KAWAHARA / 2002’.

###### Current status.

*Trox horiguchii* Ochi and Kawahara, 2002, see Pittino (2006).
